# Lipid Nanocarriers-Loaded Nanocomposite as a Suitable Platform to Release Antibacterial and Antioxidant Agents for Immediate Dental Implant Placement Restorative Treatment

**DOI:** 10.3390/pharmaceutics13122072

**Published:** 2021-12-03

**Authors:** Giuseppe Angellotti, Alessandro Presentato, Denise Murgia, Giulia Di Prima, Fabio D’Agostino, Amalia Giulia Scarpaci, Maria Cristina D’Oca, Rosa Alduina, Giuseppina Campisi, Viviana De Caro

**Affiliations:** 1 Dipartimento di Discipline Chirurgiche, Oncologiche e Stomatologiche (DICHIRONS), Università degli Studi di Palermo, 90127 Palermo, Italy; giuseppina.campisi@unipa.it; 2 Dipartimento di Scienze e Tecnologie Biologiche Chimiche e Farmaceutiche (STEBICEF), Università degli Studi di Palermo, 90123 Palermo, Italy; giuseppe.angellotti@unipa.it (G.A.); alessandro.presentato@unipa.it (A.P.); denise.murgia@unipa.it (D.M.); giulia.diprima@unipa.it (G.D.P.); amaliagiulia.scarpaci@unipa.it (A.G.S.); valeria.alduina@unipa.it (R.A.); 3 Istituto per lo Studio degli Impatti Antropici e Sostenibilità dell’Ambiente Marino, Consiglio Nazionale delle Ricerche (IAS-CNR), Campobello di Mazara, 91021 Trapani, Italy; fabio.dagostino@cnr.it; 4 Dipartimento di Fisica e Chimica, Università degli Studi Palermo, 90128 Palermo, Italy; mariacristina.doca@unipa.it

**Keywords:** nanocomposite, quercetin, ciprofloxacin, nanostructured lipid carriers, chitosan, antimicrobial, antioxidant, antibiofilm, ex vivo permeation, membrane accumulation

## Abstract

Immediate implant placement is a single-stage restorative approach for missing teeth widely used to overcome the ridge remodeling process occurring after dental extractions. The success of this procedure relies on opportune osseointegration in the surrounding tissues. To support this process, a multifunctional nanocomposite, to be applied in the fresh post-extraction socket, was here designed, prepared, and characterized. This formulation consists of quercetin (QRC)-loaded nanostructured lipid carriers (NLCs) entrapped in a chitosan-based solid matrix containing ciprofloxacin (CPX). QRC-NLCs were prepared by homogenization followed by high-frequency sonication, and thereafter this dispersion was trapped in a chitosan-based CPX-loaded gel, obtaining the nanocomposite powder (BioQ-CPX) by lyophilization. BioQ-CPX displayed desirable properties such as high porosity (94.1 ± 0.5%), drug amounts (2.1% QRC and 3.5% CPX). and low swelling index (100%). Moreover, the mechanism of drug release from BioQ-CPX and their ability to be accumulated in the target tissue were in vitro and ex vivo elucidated, also by applying mathematical models. When trapped into the nanocomposite, QRC stressed under UV light exposure (50 W) was shown to maintain its antioxidant power, and CPX and QRC under natural light were stable over nine months. Finally, both the measured antioxidant power and the antimicrobial and antibiofilm properties on *Staphylococcus aureus* demonstrated that BioQ-CPX could be a promising platform to support the single-stage dental restorative treatment.

## 1. Introduction

Implant therapy is a reliable, effective, and long-lasting replacement approach to achieve functional and aesthetic prosthetic rehabilitation of missing teeth [1,2]. Immediate dental implant placement relies on the positioning of the implant into the fresh socket after tooth extraction. In this respect, it is possible to combine surgical and restorative procedures into a single treatment [1,3,4]. This procedure accounts for several advantages, such as (i) the prevention of bone and gingival tissues resorption, (ii) high patient acceptance due to the overall reduction of trauma and in the number of surgical procedures, and (iii) short- and long-term survival rates similar to delayed implant placement [1,2,4,5]. However, some complications could be associated with immediate dental implant placement, while early implant failures can occur when osseointegration could not be achieved or is lost after implant function begins [3,5]. Osseointegration refers to a functional and organized relationship between the implant surface and the surrounding bone tissues. It is related to new bone formation around implants, allowing for paired wound healing in the implant sites [5,6,7]. Many factors can affect osseointegration after implants placement, having a significant impact on their failures, such as bone dehiscence and infection [5,6,7,8,9]. Despite the debridement of the osteotomy space aimed at removing pathogens before immediate implant placement, anaerobic infections, such as those caused by *Bacteroides* species, could always occur [10]. Indeed, implants loaded after dental avulsions could be colonized by microorganisms that might remain either intraosseous or survive in an encapsulated form, as reported in the case of *Tannerella forsythia* (former *Bacteroides forsythus*) [11]. These implant-infecting bacteria tend to form a highly structured community known as biofilm, which is enclosed and protected by an extracellular polysaccharide matrix, hard to eradicate, and adhering from the root canal to implant surfaces [12]. In addition, transient bacteria of the oral microflora (i.e., *Staphylococcus* species) are of some concern, as these microorganisms feature highly opportunistic behavior and easily develop resistance to antibiotics. Some studies reported on the occurrence of staphylococci in patients affected by periodontal disease [13] and in periimplantitis lesions, suggesting the potential role of these bacterial strains for the failing of osseointegrated dental implants [14]. Moreover, staphylococci isolated from subgingival samples can produce the exotoxin leucocidin with a killing effect towards neutrophils that migrate into the gingival area. This aspect contributes to the impairment of the host defense system, allowing periodontopathic microorganisms to elicit their pathogenicity [15]. Thus, transient microorganisms of the oral environment should not be underestimated, as they could represent a real threat and cause for the occurrence and maintenance of periodontal infections.

The first-choice material for dental implants is titanium (Ti) that, once in contact with oxygen, forms a stable layer of titanium oxide (TiO_2_) on the implant surface, playing a key role in osseointegration and corrosion prevention [16]. However, compression and frictional or torsional forces occurring on implant surfaces during the placement could damage the coating oxide layer, exposing Ti fragments and causing inflammatory responses and alterations of the epithelial cells. Moreover, as a consequence of the corrosion process, reactive oxygen species (ROS) and hydrogen peroxide (H_2_O_2_) are produced, exacerbating the inflammatory process [6].

Ciprofloxacin (CPX) is a water-soluble (logP = 0.28) fluoroquinolone antibiotic widely used in the treatment of lungs, joints, bones, airways, urinary, and endodontic infections since it shows an effective activity against both Gram-negative and -positive oral pathogens (i.e., *Staphylococcus aureus*, *Enterobacter*, and *Pseudomonas* species) [17,18,19]. In this context, the local administration of antibiotics for oral infections represents an effective approach to prevent both bacteria resistance and drug-related systemic side effects [20,21]. However, the combination of antibiotic drugs and natural antimicrobial compounds is gaining momentum as a promising strategy in different fields [22,23]. In this regard, quercetin (QRC) is a natural polyphenolic flavonoid compound abundantly found in fruits, vegetables, seeds, tea, and red wine [24], which exhibits several health-promoting properties, including antioxidant [22], anti-inflammatory [25,26], antiviral [27], anti-carcinogenic [28], and immunomodulatory actions [29]. In addition, QRC shows antimicrobial activity against both Gram-negative and -positive bacteria [24,30,31] and osteogenic effects due to osteogenic differentiation promotion towards bone mesenchymal stem cells (BMSCs) and bone loss prevention [32,33], thus underlining the importance of this natural compound in the management of dental restoration and periodontitis [34]. However, QRC features unfavorable physico-chemical properties such as poor water-solubility (logP = 1.50) and fast degradation due to alkaline pH and light exposure, resulting in it being scarcely bioavailable and difficult to manage [35,36]. A suitable and innovative approach to deliver poorly water-soluble molecules is represented by the formulation of lipid nanocarriers [37]. These systems are highly biocompatible and capable of protecting active substances from moisture, pH effect, and enzymatic activities. Moreover, lipid nanocarriers enhance drug bioavailability, release rate, and permeability through or accumulation into the physiological barriers [38]. In particular, nanostructured lipid carriers (NLCs) are composed by biocompatible solid and liquid lipids in order to obtain an imperfect lipid matrix characterized by high loading capability and able to prevent active leaching out of the encapsulated substances during storage [38,39,40]. Based on these considerations, the hydrophobic QRC could be embedded into NLCs and co-administered with the water-soluble CPX by preparing a multifunctional polymeric system. In this context, chitosan (CTS) could be the best polymer to be chosen as it is a natural water-soluble (pH ≈ 6.4, below its pKa) polysaccharide derived from the partial deacetylation of chitin found in crustaceans’ exoskeletons, insects, and fungi cell walls [41,42]. CTS consists of N-acetylglucosamine and D-glucosamine units and its physico-chemical properties depend on its molecular weight and degree of deacetylation [43,44]. Moreover, CTS is a biocompatible, biodegradable, and non-toxic polymer featuring biological activities that make it an antioxidant, mucoadhesive, and broad-spectrum antimicrobial polysaccharide [41,42]. The mechanism of action of CTS relies on the interaction between its NH_3_^+^ groups and the negatively charged moieties of the bacterial cell wall, which ultimately leads to cell lysis [43]. Recently, CTS has been widely employed as drug carrier or scaffold material for tissue engineering, regenerative medicine, and wound healing also in dentistry [41,42,44,45].

In the light of the foregoing, the aims of this study are the development, realization, and characterization of a multifunctional nanocomposite powder consisting of QRC-loaded NLCs entrapped in a chitosan matrix containing CPX and suitable for the application into the fresh post-extraction socket right before the immediate implant placement. The in situ delivery of QRC and CPX in combination with CTS should determine a synergistic multifunctional action (antibacterial, antioxidant, and anti-inflammatory) that could promote the osteointegration of dental implants loaded immediately after tooth extraction, improving their long-term survival rate.

## 2. Material and Methods

### 2.1. Materials

Quercetin dihydrate (QRC), propylene glycol, Tween 20, and PEG-18 Glyceryl Oleate/Cocoate (PEG-18 G/C) were purchased from Farmalabor (Canosa di Puglia, Italy); LipoSan ULTRA^®^ Chitosan (CTS), N-Acetylcysteine (ACys), and 18-β-Glycirrethic acid (GA) were acquired from A.C.E.F S.p.a. (Fiorenzuola d’Arda, Italy). Glyceril monostearate 40-55 (typ II) according to Ph. Eur. (GMS) and Dimethyl sulfoxide (DMSO) were supplied by Galeno srl (Comeana, Italy). Polivinilpyrrolidone K90 (PVP K90), Tween 80, Pluronic F-68, ciprofloxacin (CPX), (R)-(+)-Limonene (LIM) were purchased from Merck (Darmstadt, Germany). Beta cyclodextrin (β-CD) was purchased from Roquette Italia s.p.a (Cassano Spinola, Italy); trehalose (THR) was obtained from Hayashibara Shoji (Okayama, Japan), and glacial acetic acid was supplied by Thermo Fisher Scientific (Waltham, MA, USA). Labrasol^®^, Capryol PGMC^®^, Plurol^®^, Maisine^®^, and Labrafil^®^ M 1944 CS were kindly supplied by Gattefossé (Lyon, France). 2,2-diphenyl-1-picrylhydrazyl 95% (DPPH) was purchased from Alfa Aesar (Haverhill, MA, USA).

Phosphate buffer saline (PBS) pH 7.4 was prepared by dissolving 4.03 g of NaCl, 0.10 g of KCl, 0.095 g of KH_2_PO_4_, and 0.76 g of Na_2_HPO_4_·2H_2_O in 0.5 L of distilled water.

Citrate buffer (pH 5.5) was prepared by dissolving 1.026 g of tri-sodium citrate dihydrate and 0.318 g of citric acid monohydrate in 0.5 L of distilled water.

The isotonic solution was prepared by dissolving 9 g of NaCl in 1 L of distilled water, while the isotonic saline solution containing trehalose was prepared adding 5 g of trehalose in 1 L of abovementioned solution.

The employed chemicals (A.C.E.F S.p.a., Fiorenzuola d’Arda, Italy) and solvents (VWR International, Milan, Italy) were of analytical grade and were used without further purification. Animal tissue specimens withdrawn from pigs intended for human consumption were kindly supplied by the Municipal Slaughterhouse of Villabate (Palermo, Italy).

### 2.2. Methods

#### 2.2.1. Preliminary Studies

##### QRC Stability Assay in Aqueous Environments

QRC degradation rate was investigated in: (i) PBS pH 7.4 containing 10% v/v of DMSO (solvent A) and (ii) citrate buffer pH 5.5 solution amended with either 10% v/v of DMSO (solvent B) or 10% v/v of DMSO and 0.002% w/v of ACys (solvent C). The prepared QRC aqueous solutions (0.01 mg/mL) were kept at room temperature for 6 h in quartz cuvette located in the UV–Vis spectrophotometer (Shimadzu 1700 instrument, Kyoto, Japan). At scheduled time intervals (10 min), QRC spectra were recorded (from 200 to 700 nm) to highlight any changes in terms of sharp and absorption peaks. Results were reported as absorbance variation at lambda max (360 nm) over time according to the following equation:

$$\mathrm{Abs}\;\mathrm{variation}\;\%=\frac{{\mathrm{Abs}}_{\mathrm{tn}}}{{\mathrm{Abs}}_{t0}}\times 100$$

where Abs_t0_ and Abs_tn_ are the QRC absorbance recorded initially and at each time point, respectively. The stability assays were performed in triplicate. Results are reported as means ± SE.

##### QRC and CPX Solubility Studies in Aqueous Media

QRC solubility was tested in citrate buffer pH 5.5 with 0.002% w/v of ACys supplemented with DMSO 10% (v/v), either 2 or 3% (w/v) of β-CD. QRC, in excess, was added to each medium (1 mL) until precipitation occurred. The obtained suspensions were kept under constant stirring at 37 ± 0.5 °C (Heidolph MR3001K Hotplate Stirrer with Heidolph EXT3001 Temperature Probe, Heidolph Instruments, Schwabach, Germany) for 120 min. Suspensions were then centrifuged for 15 min at 12,500 rpm (microcentrifuge mySPIN™ 12 Mini Centrifuge, Thermo Fisher Scientific) and the supernatants were successively withdrawn, filtrated through a 0.22 µm hydrophilic PTFE filter (Millipore, Burlington, KS, USA), diluted with methanol and spectrophotometrically analyzed to quantify the amount of QRC using the appropriate blank and calibration curve. As for CPX, its solubility, concentration, and spectral features were evaluated using citrate buffer pH 5.5 amended with 3% of β-CD in the presence/absence of acetic acid 0.1 % v/v, following the procedure earlier described. The supernatants collected after centrifugation were diluted with citrate buffer pH 5.5 and subjected to UV–Vis analysis to determine the amount of dissolved CPX. The solubility tests were performed in triplicate. Results are reported as means ± SE.

#### 2.2.2. Preparation and Characterization of Nanostructured Lipid Carriers (NLCs)

##### Screening of the Liquid Lipids

Labrasol^®^, Capryol PGMC^®^, Plurol^®^, Maisine^®^, Labrafil^®^ M 1944 CS, and PEG-18 G/C were selected as liquid lipids to evaluate their ability to completely dissolve the desired amount of QRC (5% w/w). Briefly, 50 mg of QRC was added to 950 mg of each lipid. Each mixture was then ultrasonicated (Emerson Branson 1200, Branson, Brookfield, USA) for 15 min, allowing QRC powder disaggregation. Then, mixtures were maintained under magnetic stirring (200 rpm) and kept in a silicone thermostatic bath heated up to 60 °C (Heidolph MR3001K, Schwabach, Germany) for 30 min. After cooling at room temperature, samples were evaluated in terms of appearance and clearness.

##### Evaluation of the Lipid Mixtures

QRC (50 mg) was initially dispersed in the appropriate amount of Labrasol^®^ and then added to GMS and GA at various ratios (Table 1), while increasing the temperature up to 120 °C and maintaining the mixtures under constant stirring until a clear dispersion was formed. Subsequently, the obtained clear mixtures were rapidly cooled down at 10 °C within 10 min and stored at 4 °C until the next use. Accordingly, QRC-free mixtures (blank) were prepared, featuring MIX 4 and MIX 5 excipient ratios, as reported in Table 1. The melting point of each mixture was determined by using a melting point apparatus Stuart Melting Point Apparatus SMP30 and are uncorrected. In brief, samples were placed in a capillary glass and analyzed until complete melting occurs (heating rate: 5 °C/min). The analyses were performed in triplicate. Results are reported as means ± SE.

##### Preparation of NLCs

The QRC-loaded NLCs (QRC-NLCs) and QRC-free NLCs (NLCs) were prepared according to the homogenization followed by high-frequency sonication method [46] by distinctly preparing the lipid and the aqueous phases. Briefly, 160 µL of LIM was added to 800 mg of the selected melted lipid phases (MIX 4, MIX 5, Blank 1, and Blank 2). Simultaneously, the aqueous phase was prepared by dissolving Tween 80 (2% w/v), Pluronic F-68 (2% w/v), and ACys (0.002% w/v) in citrate buffer pH 5.5. The two phases were separately heated up to 80 °C and kept at this temperature for 5 min. Subsequently, 40 mL of the prepared aqueous phase were slowly added to the lipid phase and homogenized by Ultraturrax (Polytron Model PT MR 2100, Kinematica, Malters, Switzerland) at 19,000 rpm for 1 min. The resulting hot melt coarse emulsion was sonicated (20 kHz) with cycles of 0.7 s of activity (on) and 0.3 s of inactivity (off) for a total of 15 min. This procedure was repeated twice, firstly at room temperature and subsequently by placing the samples into an ice/water/NaCl bath, allowing solidification of the NLCs. Since MIX 4 and Blank 1 feature the same lipid ratios, these samples are indicated as QRC-NLC-A and NLC-A, respectively. Similarly, MIX 5 and Blank 2 are termed as QRC-NLC-B and NLC-B.

##### Quercetin (QRC) Quantification: Drug Loading (DL%) and Loading Efficacy (LE%)

Each QRC-loaded NLC sample was tested for drug loading (DL) and loading efficacy (LE) percentages determination. Firstly, the amount of QRC (*QRCtot*) was determined through UV–Vis spectrophotometer by diluting (200 folds) the NLCs in methanol. Consequently, LE% and DL% were indirectly determined by the ultrafiltration method. Specifically, a 100 folds dilution of the NLCs in distilled water was inserted in the upper chamber of a centrifuge filter tube (Ultrafree-MC, Millipore, Burlington, MA, USA, cut off 0.2 µm) and centrifuged at 4000 rpm for 2 min. The solution in the bottom chamber of the filter tube was collected and analyzed by HPLC to determine the unencapsulated amount of QRC (*QRCout*). Therefore, DL and LE percentages were calculated according to the following equations:

$$\mathrm{DL}\%=\frac{\left(\mathrm{QRCtot}-\mathrm{QRCout}\right)\;\left(\mathrm{mg}\right)}{\mathrm{Wlip}\;\left(\mathrm{mg}\right)}\times 100$$

$$\mathrm{LE}\%=\frac{\mathrm{QRCtot}-\mathrm{QRCout}\;\left(\mathrm{mg}\right)}{\mathrm{QRCtot}\;\left(\mathrm{mg}\right)}\times 100$$

where Wlip is the amount of NLCs components mass (mg). The analyses were performed in triplicate and results were reported as means ± SE.

##### Dynamic Light Scattering (DLS) and Z-Potential Analysis

DLS and Z-Potential measurements were performed at 25 ± 0.5 °C by using a Malvern Zetasizer NanoZS instrument equipped with a 532 nm laser at a fixed scattering angle of 173°. Aqueous dispersions of NLC (0.2 mg/mL) were prepared and analyzed in triplicate. Moreover, dispersions of the employed surfactants were used as control samples.

##### QRC-NLCs Scanning Electron Microscopy (SEM)

The morphological analysis of the QRC-NLC-A sample was performed by using a Zeiss EVO MA10 (Zeiss, Oberkochen, Germany) scanning electron microscope equipped with a SE-Everhart-Thornley secondary electron detector with lanthanum hexaboride (LaB6) cathode as the source of electrons, an accelerating voltage of 20 keV, and probe voltage of 10 pA. Electron micrographs were acquired in a high-vacuum condition (HV, about 10^−7^ mbar) and magnified up to 200 nm. In order to increase the surface electrical conductivity of the QRC-NLC-A sample, a few drops of the NLC dispersion were put on an aluminum stub and placed in a CaCl_2_ desiccator at 4 °C for 24 h. Afterward, the sample was coated with an ultrathin layer of gold (thickness about 2 nm) with an AGAR Sputter Coater type system, prior observations.

##### Preparation of QRC-NLC-CPX and NLC-CPX

MIX4 samples was chosen to prepare the NLC dispersions enriched with the selected antibiotic. CPX was dissolved in citrate buffer pH 5.5 supplemented with 0.1% (v/v) of glacial acetic acid and the resulting solution (10 mg/mL) was properly diluted with the previously described complete aqueous phase (final CPX concentration: 2 mg/mL). Then, the NLCs were prepared as described above (Section 2.2.2—Preparation of NLCs) by using the prepared CPX-loaded aqueous phase. The obtained NLCs dispersed in the CPX-containing aqueous phase were indicated as QRC-NLC-CPX. The blank sample was also prepared in the same manner using Blank 1 lipid ratio and marked as NLC-CPX. Samples were subjected to DLS and Z-potential measurements as described above.

#### 2.2.3. Preparation and Characterization of the Nanocomposite

##### Preparation of the Nanocomposites

CTS (3% w/v) and PVP K90 (0.3% w/v) were dispersed in pre-acidified (pH 4 by acetic acid) distilled water at room temperature under continuous stirring (800 rpm) until a dense and homogeneous gel was obtained. Subsequently, it was stored at 4 °C for 24 h before use. To prepare the nanocomposite, 10.67 g of gel and 320 mg of THR, used as a cryoprotectant, were added to 40 mL of the NLC dispersion. Samples were then homogenized at 12,000 rpm for 1 min into an ice/water/NaCl bath. The obtained nanocomposites were stored at −80 °C overnight and then freeze-dried (Labconco FreeZone^®^ 2.5 Liter Freeze Dry System, KS, USA). Table 2 reports the list of nanocomposites, while the “Blank matrix” was prepared by freeze-drying the above-described CTS gel.

##### Porosity Determination

Porosity of the BioQ-CPX was determined mathematically [47]. Firstly, the theoretical volume (Vt), meant as the sum of the volumes of each component constituting the sample, was calculated according to the following equation:

$$\mathrm{Vt}=\sum \frac{m_{n}}{\rho_{n}}$$

where m and ρ are mass and density of each component, respectively.

After the freeze-drying process, the real volume (Vr) of the nanocomposite was determined by placing the sample in a graduated cylinder and measuring the occupied volume (length × width × height). Then, porosity was determined according to the following equation:

$$\mathrm{Porosity}\%=\frac{\mathrm{Vr}-\mathrm{Vt}}{\mathrm{Vt}}\times 100$$

The determination of the real volume was performed in triplicate.

##### Determination of QRC and CPX Amounts into the Nanocomposites

Three samples (5 mg) were taken from different parts of either BioQ-CPX or BioNLC-CPX nanocomposites, dissolved in 20 mL of methanol, and analyzed by UV–Vis spectrophotometer to quantify QRC (when present) and CPX. Each experiment was performed in triplicate for each batch of nanocomposite developed. Results are expressed in terms of DL% and reported as means ± SE.

##### Scanning Electron Microscopy (SEM)

To evaluate the morphology and the topographic characteristics of BioQ-CPX, SEM analyses were performed as described above. Each sample was coated with an ultrathin layer of gold (thickness about 2 nm) with an AGAR Sputter Coater type system.

##### Swelling Test

To investigate the swelling properties of BioQ-CPX, mini tablets were obtained as previously reported [46] by applying a pressure of 1 ton/cm^2^. The swelling test was carried out by placing a mini tablet on a microscope slides and weighting at t = 0 (Wd) using an analytical balance (Mettler, Columbus, OH, USA, Mod. AE 240). Every 5 min for 1 h, 0.5 mL of citrate buffer solution pH 5.5 was placed on the mini tablet to soak it. Then, at each time point, the excess of medium was gently removed using a filter paper and the hydrated mini tablet weight (Wh) was evaluated. The water uptake, expressed as swelling index, was determined according to the following equation:

$$\mathrm{SI}\%=\frac{\left(\mathrm{Wh}-\mathrm{Wd}\right)}{\mathrm{Wd}}\times 100$$

The experiments were performed on six mini tablets and results are reported as means ± SE.

##### Hygroscopicity Studies

The hygroscopicity of the BioQ-CPX nanocomposite was analyzed using an analytical five decimal balance (Mettler, Mod. AE 240). Before each analysis, a previously dried desiccant silica box was kept inside a drying oven (StabiliTherm, Thermo Scientific) for 10 min to remove any trace of humidity into the weighing chamber. Aliquots of the dry sample were placed on a microscope slide and accurately weighed. Afterward, the silica box was removed and both the doors of the balance were kept open, allowing the contact with the environmental moisture (relative humidity (RH): 60%). At scheduled time intervals (10 min), the slide doors were closed and the weight of the analyzed sample was recorded. Sample weight variation was calculated as follows:

$$\mathrm{Weight}\;\mathrm{increase}\;\%=\frac{\mathrm{Wt}-\mathrm{Wi}}{\mathrm{Wi}}\times 100$$

where Wt and Wi refer to the weight of nanocomposite at time t and the initial weight of dry powder, respectively. Data are reported as means ± SE (*n* = 6).

##### Drug Release Studies

The ability of the BioQ-CPX nanocomposite to release both CPX and QRC was separately assessed in vitro.

The CPX release profile was evaluated by performing two different methods using: (a) pre-activated dialysis tubes (Spectra/Por^®^ Standard Grade Regenerated Cellulose, MWCO 12–14,000 Da, Thermo Fisher Scientific) filled with 1 mL of BioQ-CPX (10 mg/mL) dispersed in citrate buffer pH 5.5 amended with glacial acetic acid 0.1% (v/v). The system was submerged in 15 mL of the same medium; (b) Franz diffusion cells (Permeagear, flat flange joint, 9 mm orifice diameter, 15 mL acceptor volume, SES GmbH-Analysesysteme, Bechenheim, Germany) consisting in a donor chamber filled with 10 mg of powder and soaked with 0.5 mL of citrate buffer pH 5.5, and an acceptor chamber filled with 15 mL of the same medium plus glacial acetic acid 0.01% (v/v). A cellulose dialysis membrane (Medicell Membranes, London, UK: MWCO: 12–14,000 Da) previously soaked with the acceptor fluid, was inserted between the two compartments.

QRC release studies were carried out using a modified transwell permeable support (0.45 µm) filled with 5 mg of the BioQ-CPX powder and soaked with 0.2 mL of citrate buffer pH 5.5. The entire system was dipped inside a beaker filled with 8 mL of 1-octanol as a suitable acceptor fluid. At scheduled time intervals, 0.5 (for CPX release experiments) or 1 mL (for QRC release experiments) samples were collected, and drug amount was determined by UV–Vis analyses. The experiments were carried out at 37 ± 0.5 °C under constant stirring and protected by light exposure while, immediately after sampling, opportune volumes of fresh acceptor fluid were added to maintain the sink conditions. Data are reported as means ± SE (*n* = 6). Drug release profiles were elaborated using OriginPro 8.5 software and fitted with semi-empirical model equations. Fittings were validated by using R^2^ value where a p-value of less than 0.05 was considered statistically significant.

##### Tissue Preparations

Tissue specimens were obtained from the vestibular area of the retromolar trigone derived from 6–8-month-old pigs supplied by the Municipal Slaughterhouse of Villabate (Palermo, Italy) since withdrawn from animals intended for human consumption. By a refrigerated transport box, the specimens were immediately transferred to the laboratory within 1 h from animal sacrifice and surgically treated to remove both adipose and connective tissues in excess. Afterward, they were dipped for 30 min in a 5% THR (w/v) isotonic solution and then stored at −80 °C for a minimum period of one week. Before the permeation/penetration experiments, tissue samples were accurately washed to remove the cryoprotectant, and, subsequently, thermal shocked to obtain the buccal mucosa. Briefly, tissue samples were dipped for 30 s in a pre-warmed (70 °C) isotonic solution, and then the mucosa was carefully peeled off from the connective tissue, obtaining the heat-separated epithelium along with the intact basal lamina [48]. Finally, it was equilibrated in an isotonic solution for 3 h by replacing the medium every 15 min to remove all the biological matter, which could affect the analysis, before use.

##### Ex Vivo Permeation/Penetration Experiments

The ex vivo permeation/penetration studies were carried out using vertical Franz-type diffusion cells. Briefly, appropriate sections of buccal mucosa were mounted between the donor and the acceptor chambers filled with 1 mL of citrate buffer pH 5.5 and 15 mL of citrate buffer pH 5.5 amended with acetic acid 0.1% (v/v), Acys 0.002% (w/v), and β-CD 3% (w/v). The whole system was equilibrated at 37 ± 0.5 °C for 30 min. Afterward, the medium was removed from the donor compartment, promptly replaced with 30 mg of BioQ-CPX, and soaked with 0.2 mL of citrate buffer pH 5.5. Every 30 min, aliquots (0.5 mL) were withdrawn from the acceptor chamber and immediately replaced with the same volume of fresh acceptor fluid to maintain the sink conditions. Experiments were carried out at 37 ± 0.5 °C in the dark and under constant stirring, being stopped at different time points ranging from 1 to 6 h. The amount of permeated QRC and CPX were quantified by UV–Vis analysis. Data are reported as means ± SE (*n* = 6).

##### Quantification of QRC and CPX Entrapped into the Buccal Mucosa

At the end of each permeation experiment, the amount of QRC and CPX entrapped into the porcine buccal mucosa was evaluated at different experimental end points (from 1 to 6 h). Franz cells were disassembled, while the buccal mucosa was washed with PBS pH 7.4 and subjected to QRC and CPX extraction. Briefly, the mucosa was dipped into 2 mL of methanol and warmed up to 60 °C. The extraction procedure was performed twice, and the collected extraction liquors were transferred into a 10 mL flask and brought to volume with fresh methanol. Thus, QRC and CPX amounts were determined by UV–Vis analysis. Results are reported as means ± SE.

#### 2.2.4. Evaluation of the Nanocomposite Antioxidant Activity

To evaluate the antioxidant activity of BioQ-CPX, the 2,2-diphenyl-1-picrylhydrazyl (DPPH) assay was performed [49]. Accurately weighted aliquots of the nanocomposite were put in a volumetric flask and dissolved in methanol in order to obtain nanocomposite solutions having concentration of 4, 2, 1, and 0.5 mg/mL. A measure of 0.5 mL of nanocomposite solution was transferred in polystyrene cuvette, added to 2.5 mL of DPPH ethanol solution (0.0295 mg/mL), and preserved by light exposure using foil. At determined time intervals (0, 0.25, 1, and 2 h), the DPPH concentration was measured spectrophotometrically at room temperature by observing the spectral range 400–800 nm and selecting λ_max_ = 515 nm for quantification. The DPPH calibration curve was constructed analyzing DPPH ethanol solutions in the concentration range of 0.039–0.0156 mg/mL (λ_max_ = 515 nm, regression equation: Abs = 27.15 + 0.048 × [DPPH mg/mL], R = 0.999).

Similarly, the assay was carried out both on BioNLC-CPX samples (4, 2, and 1 mg/mL) used as negative control and QRC solutions (0.08, 0.04, 0.02, and 0.01 mg/mL) used as positive control. The analyses were performed in triplicate and results are reported as percentage of residual concentration of DPPH (%RCD) radical form according to the following equation:

$$\%\mathrm{RCD}\;=\left(\frac{{\mathrm{DPPH}}_{i}-{\mathrm{DPPH}}_{t}}{{\mathrm{DPPH}}_{i}}\right)\times 100$$

where DPPH_i_ and DPPH_t_ are the concentration of radical DPPH at t = 0 and at each time point, respectively. Results are expressed as means ± SE.

#### 2.2.5. Microbiological Evaluation of the Nanocomposite

Kill curve assays were performed to test the antimicrobial efficacy of BioQ-CPX, BioQ, and the Blank CTS matrix against the indicator pathogen *Staphylococcus aureus* ATCC 25923 strain, growing as planktonic cells [50]. Briefly, a single colony of *S. aureus* strain was picked up and pre-cultivated (ca. 16 h) at 37 °C with shacking (160 rpm) in tryptone soy broth medium (hereinafter named as TSB; Merck, Milan, Italy). The same medium was solidified by adding 15 g/L of bacteriological agar when needed. Bacterial cells were then inoculated [0.05% v/v corresponding to ca. 5 × 10^5^ colony forming units (CFU) per mL of culture] in TSB medium amended with increasing concentrations (i.e., 1, 2.5, and 5 mg/mL) of the above-mentioned samples. The set up bacterial cultures were challenged for 24 h according to the same pre-culturing conditions. The actual number of viable *S. aureus* CFU/mL—who survived the challenge exerted by BioQ-CPX, BioQ, and Blank matrix—was evaluated through the spot plate count method and compared to unchallenged cultures. The data are reported as average values (*n* = 3) of the CFU/mL in the logarithmic (Log_10_) scale with standard deviation (SD).

BioQ-CPX and CPX were also tested for their ability to inhibit *S. aureus* cells growing as a biofilm. Briefly, pre-cultivated *S. aureus* cells for 16 h at 37 °C were inoculated (1% v/v corresponding to ca. 1 × 10^7^ CFU/mL) in TSB medium. The set-up culture was aliquoted (200 μL) in a 96-well microtiter plate. Afterward, each bacterial culture (*n* = 3) was amended with 5 mg of BioQ-CPX prepared as described above (Swelling test—Section 2.2.3), having a diameter size of ca. 5 mm, while CPX was added as liquid solution at the same concentration featuring the BioQ-CPX formulation. The microtiter plate was then incubated for 24 h at 37 °C under static mode, allowing for bacterial biofilm formation. After the incubation period, bacterial cultures have been disposed, while each well was washed 3 time with 200 μL of sterile physiological (NaCl 0.9% w/v) solution to remove loosely adherent cells. Then, *S. aureus* biofilms were stained by adding 200 μL of an aqueous crystal violet (0.1% w/v) solution at room temperature for 15 min. Subsequently, wells were washed 5 times with 200 μL of physiological solution to remove the excess of unbound crystal violet and left to dry for 20 min at 65 °C. Finally, crystal violet was solubilized by adding 200 μL of an acetic acid (30% v/v) solution, the absorbance (Abs) being read at 600 nm using a DU-730 Life Science spectrophotometer (Beckman Coulter, Brea, CA, USA). Unchallenged cultures were used as reference biofilms (positive control), while wells containing only the medium utilized in this study were used as a negative control. The percentage of biofilm inhibition was calculated as follows:

$$\mathrm{Biofilm}\;\mathrm{inhibition}\;\left(\%\right)=\frac{\left(\mathrm{Abs}\;\mathrm{positive}\;\mathrm{control}-\mathrm{Abs}\;\mathrm{challenge}\right)}{\mathrm{Abs}\;\mathrm{positive}\;\mathrm{control}}\times 100$$

#### 2.2.6. Stability Evaluations of Nanocomposite

##### Evaluations of QRC Stability in BioQ-CPX after UV Irradiation by EPR Spectroscopy

EPR measurements were carried out on 25 mg of QRC and BioQ-CPX at room temperature using a Bruker ELEXSYS E-500 spectrometer (Bruker, Billerica, MA, USA) operated at 9.8 GHz (X-band), operating at 6 mW microwave power and 0.15 mT modulation amplitude.

To evaluate the effects of light exposure in the samples, UV-A/UV-B irradiator alternatively equipped with lamps of 25 W and 50 W (Light-Emitting Diode Philips, Amsterdam, The Netherlands) was used.

Preliminarily, the paramagnetic center of QRC (pure drug) before and after 20 min of UV irradiation (50 W) was characterized.

Afterwards, to evaluate the BioQ-CPX capability in preserving QRC from light exposure, aliquots of QRC and BioQ-CPX were irradiated by 25 W or 50 W lamps over time. In particular, each sample was spread in a glass holder to obtain a thickness less than 5 mm so as to achieve a good irradiation distribution. UV irradiation was accomplished up to 60 min for all samples and EPR analysis was carried out every 5 min. Signals were normalized to the QRC mass contained in each sample. Data are reported as mean ± SE (*n* = 3).

##### Evaluation of CPX and QRC Stability over Time by Quantitative Analyses

BioQ-CPX capability of preserving the integrity of bioactive molecules was evaluated over time by quantitative determination of CPX and QRC. Immediately after preparation (t = 0), randomly selected and accurately weighted aliquots (*n* = 3) of the same batch of nanocomposite were dissolved in methanol and subjected to UV–Vis analyses to evaluate the amount of QRC and CPX contained, that were considered as 100%. Afterward, the nanocomposite was stored at room temperature and under natural light exposure. At scheduled time intervals (for a total of 9 months), aliquots (*n* = 3) were withdrawn and evaluated both in terms of amount of active compounds as well as in terms of spectrum shape by UV–Vis analysis. Data are reported as CPX and QRC percentage variation at each time point compared to the starting value (100%). Stability test was carried out on three batches of nanocomposite and the results are reported means ± SE.

#### 2.2.7. Quantitative Analysis of Drugs

##### By UV–Vis Analysis

To evaluate QRC and CPX amounts entrapped in NLCs dispersion, nanocomposites, porcine membranes, and in dissolution media, UV–Vis analyses were performed using a Shimadzu 1700 instrument (Kyoto, Japan) with the appropriate calibration curve and blank. The linearity of the analytical procedure was statistically determined by regression analysis of five concentrations in the range below specified and evaluated in triplicate. Known concentrations of the drug were added in the solution and UV analyzed to check the accuracy of the method. Three different concentrations covering the examined range were selected and analyzed in triplicate for the recovery. By calculating relative standard deviation (%RSD) of the mean recoveries, the precision of the methods was defined. UV methods were simple, accurate, and reproducible.

For QRC quantification, two calibration curves were performed: in methanol at λ_max_ = 370 nm, in the linearity range of 0.0006–0.05 mg/mL, regression equation was Abs = −0.00224 + 44.36 x [mg/mL], (R = 0.999, SE 0.0059) and in 3% β-CD (w/v) citrate buffer pH 5.5: at λ_max_ = 370 nm, in the linearity range of 0.0001–0.005 mg/mL; regression equation was Abs = −0.00461 + 64.57 x [mg/mL], (R = 0.999, SE 0.0026).

For CPX quantification, two calibration curves were performed: in methanol at λ_max_ = 282 nm in the linearity range of 0.0005–0.01 mg/mL; regression equation was Abs = −0.00646 + 119 x [mg/mL], (R = 0.999; SE 0.0054); and in 3% β-CD (w/v) citrate buffer pH 5.5 at λ_max_ = 276 nm, in the linearity range of 0.0005–0.008 mg/mL; regression equation was Abs = −0.0355+106 x [mg/mL]; (R = 0.999, SE 0.0048).

No interferences between drugs and components of formulations were observed at the testing concentrations and no change at λ_max_ of drug absorbance was experienced in the presence of each excipient. Intraday and interday variations observed during the collection of experimental data were lower than sensibility.

##### By HPLC Analysis

HPLC analysis was performed to quantify QRC by using a HPLC Agilent Instrument 1260 Infinity equipped with a Quaternary Pump G1311B, a Diode Array Detector 1260 Infinity II, an autosampler, a column oven, and a computer integrating apparatus (OpenLAB ChemStation 3D UV Workstation, Santa Clara, CA, USA). Chromatographic separation was achieved by a reversed-phase column Ace^®^ Excel Super C18 (5U, 100A, size 125 × 4.60 mm thermostated at 25 ± 1 °C; injected volume 20 µL) and employing a 0.1% (v/v) TFA solution in water (solvent A) and acetonitrile (solvent B), according to the following time program: 0–2 min A:B = 70:30; 2–8 min A:B = 20:80; 8–12 min A:B = 70:30. The flow rate was set at 1 mL/min, the DAD at UV wavelength from 200 to 700 nm and the lambda at 360 nm was selected for QRC quantification. In these conditions, QRC retention time was 5.89 min. Standard curve was used for the quantification of integrated areas under the peaks. The calibration curve was performed in the concentration range of 0.0002–0.01 mg/mL by injecting six standard solutions of QRC in methanol. HPLC data were highly reproducible and linearly related to concentration (y = 18321.5 x −1.144; R = 0.999). LOD and LOQ were 0.0006 µg/mL and 0.002 µg/mL, respectively.

#### 2.2.8. Data Analysis

Data were expressed as mean ± standard error (SE). All differences were statistically evaluated by the Student’s *t* test or the one-way analysis of variance (ANOVA or F-test) with the minimum levels of significance with *p* < 0.05.

## 3. Results and Discussion

### 3.1. Preliminary Evaluations: QRC and CPX Solubility and Stability

QRC is a natural polyphenolic compound which is easily susceptible to chemical degradation induced by temperature, pH, and light exposure [51]. As it is crucial that QRC remains chemically stable during handling, its stability in aqueous media was firstly evaluated by UV–Vis spectrophotometry, analyzing QRC solutions (0.01 mg/mL) either in PBS 7.4 pH or citrate buffer 5.5 pH, which were amended with 10% v/v DMSO as a co-solvent to improve QRC solubility in order to prepare easily detectable solutions.

No changes in the shape of the absorption spectra were observed for the analyzed solutions, while a decrease of the maximum peak at λ 360 nm was highlighted over time.

**Figure 1:**
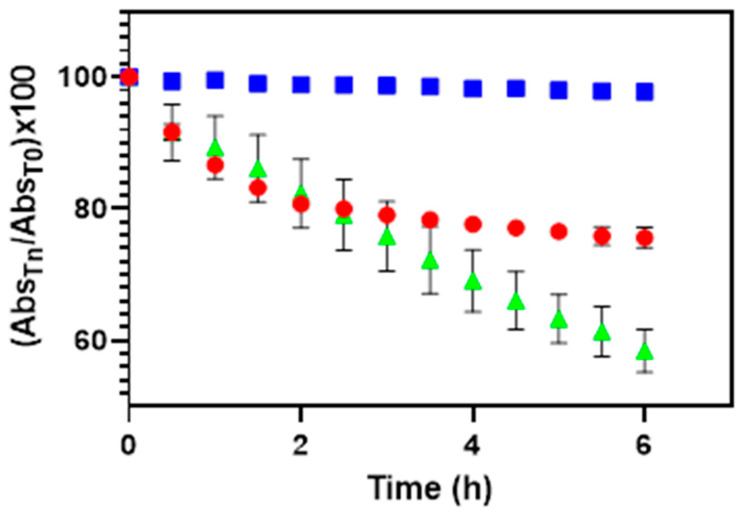
Stability evaluation of QRC expressed as percent of absorbance variation vs. time in: PBS 7.4 pH + DMSO (▲); citrate buffer 5.5 pH plus DMSO (●); citrate buffer 5.5 pH plus DMSO and ACys (■).

To better compare the behavior of QRC solutions in different solvents, variations in terms of absorbance value (at λ_max_ = 360 nm) over time were expressed as percentage of QRC absorbance variation with respect to its absorbance value at time = 0. After 6 h, this value falls in both cases. However, some relevant differences were highlighted as the absorbance ratio exhibit a higher decrease in the PBS solution instead of the citrate buffer one (42% vs. 25%). These data confirm that neutral/low alkaline pH values negatively affect QRC stability [51]. Consequently, citrate buffer pH 5.5 was selected for further evaluations to obtain a suitable aqueous medium able to stabilize QRC. Due to the well-known antioxidant properties of ACys, 0.002% w/v of the latter was added to the citrate buffer, unveiling how QRC stability was improved over time (Figure 1). Thus, this medium was selected as the best one for further preparation steps/studies (e.g., as aqueous medium for the preparation of lipid nanoparticles).

Moreover, as the poor water solubility of QRC [52] could negatively affect the evaluation of its permeation in the ex vivo experiments due to the lack of sink conditions and the absence of a concentration gradient representing the main driving force for the passive diffusion of the drug, citrate buffer pH 5.5 (supplemented with ACys 0.002% w/v) containing DMSO or β-CD as solubilizing agents was tested to increase the QRC solubility (Table 3).

Results showed that QRC solubility was greatly enhanced in presence of either DMSO (20-fold) or β-CD (70- and 42-fold for 3% and 2%, respectively) when compared to its well-known very low water solubility. Thus, the citrate buffer pH 5.5 together with ACys and 3% β-CD was chosen as aqueous medium to enhance QRC solubility and stability as often as it was needed in subsequent studies (e.g., as acceptor fluid for the ex vivo experiments). On the other hand, as the main aim of this work is the administration of QRC together with CPX, also the antibiotic solubility should be considered. Since CPX is a water-soluble molecule (logP = 0.28), to maximize its solubility in the selected medium, acetic acid 0.1% v/v was further added, resulting in enhanced antibiotic solubility over 100 mg/mL.

### 3.2. Preparation and Characterization of Empty and QRC-Loaded NLCs

Nanostructured lipid carriers (NLCs) are lipid-based nanosystems able to interact with the lipid components of the biological tissues increasing the bioavailability of poorly water-soluble molecules such as QRC. NLCs overcome the limitations of solid lipid nanoparticles (SLNs), consisting of unsatisfactory drug loading and short shelf-life due to the composition comprising a single solid lipid excipient. NLCs are formulations containing at least two lipids (both liquid and solid lipid), thus resulting in advantages mainly due to the amorphous and disordered structure of the lipid core [53]. For NLCs development, the crucial step is lipids screening, which will determine the physio-chemical stability and bioavailability of both the drug and the carrier. Moreover, all the excipients used for NLCs preparation must be non-toxic, hypoallergenic, and chemically inert [54].

Here, glyceryl monostearate (GMS), a natural mixture of monoacylglycerols together with variable amounts of di- and triacylglycerols, was chosen as principal solid lipid constituent of NLCs, due to its good surfactant properties as well as its high biocompatibility, as it is a Ph. Eur. and FDA-approved excipient for pharmaceutical applications. To carefully choose the most suitable liquid lipid component, the capability of dissolving the desired amount of QRC (5% w/w) was selected as leading parameter by observing the appearance of the resulting QRC–liquid lipid mixture in terms of clearness, as it indicates the absence of any residual QRC solid particle into the blending. All the tested liquid excipients were chosen due to their biocompatibility and biodegradability; moreover, they all feature one or more PEGylated functions conferring them the amphipathic nature needed to solubilize QRC. QRC–liquid lipid mixtures were prepared at a fixed drug-to-excipient ratio (5:95). Table 4 reports the obtained appearance data for all the prepared mixtures.

As expected, Labrasol^®^, the PEGylated (PEG-8) medium chain fatty acid triglyceride of capric and caprylic acid was the most capable of dissolving the desired amount of QRC, being in line with its emulsifying properties, which make this lipid widely used in both the pharmaceutical and cosmetic fields [55,56]. Consequently, Labrasol^®^ was selected as liquid lipid and mixed with the other solid lipids chosen for this study. Moreover, 18-β-glycirrethic acid (GA), a pentacyclic triterpenoid deriving from the hydrolysis of glycyrrhizic acid, was also employed due to its anti-inflammatory, antiviral, and antimicrobial properties that make it widely used in the treatment of several oral diseases [57,58]. Firstly, QRC solubilization in GMS at 120 °C in ratio 5:95 was proven, highlighting that GMS alone was not able to form a clear dispersion and, for prolonged time, the high temperature required led to the mixture browning due to QRC decomposition. Thus, the solid and liquid components were blended at various ratios (MIX), as reported in Table 5, by hot treatment (120 °C) following a quick cooling down to promote QRC solidification in the mixture in amorphous form [59]. Finally, each MIX was evaluated in terms of appearance when melted as well as in terms of melting point temperature (Table 5). In particular, the mixture melting point is the parameter that determines both NLC storage and efficacy. Ideally, on one hand, the melting point should not be too low to avoid NLCs melting at the storage temperature. On the other, it should also not be too high since the resulting nanocarriers must melt once in contact with the tissues of oral cavity, thus allowing drug released. Hence, the goal is to obtain lipid nanoparticles able to soften once applied on the target site and promote QRC absorption by interaction with the lipid components of the administration tissue.

As a result, high GA percentage (MIX 2) turned brown and opalescent, likely due to QRC degradation related to the high temperature kept for a prolonged time (necessary to melt the GA over 5%). Moreover, Labrasol and GMS at lipid ratio of 50:50 (i.e., MIX 1 and MIX 3) was not able to dissolve the whole amount of QRC, while the ratio 25:75 (i.e., MIX 4 and MIX 5) allowed for a quick QRC solubilization thus avoiding temperature-related degradation phenomena.

In light of these results, MIX 4 and 5 were those resulting homogeneous, bright yellow in color (likely due to QRC), and once cooled they showed a smooth and flawless surface. Consequently, MIX 4 and 5 were selected to prepare QRC-NLCs and the corresponding blank samples, named Blank 1 and Blank 2, respectively; these last resulted homogeneous, white in color, and having a lower melting point temperature, likely due to the absence of QRC, which contributes (due to its m.p. of 316 °C) to raising the m.p. of the mixtures. To the mixtures, intended for preparation of NLCs, (R)-(+)-Limonene (LIM) was added, allowing for a better QRC absorption into mucosal tissues, which is in line with the well-known properties of LIM as a chemical penetration enhancer [60], as well as contributing to the antimicrobial activity to NLCs [61].

As external aqueous medium for NLCs preparation, ACys, Tween 80, and Pluronic F-68 were dispersed in the citrate buffer pH 5.5. ACys and citrate buffer were chosen to stabilize QRC, while Tween 80 and Pluronic F-68 to reduce the interfacial tension between the lipid and aqueous phases thus stabilizing the system, as previously reported [62].

A hot pre-emulsion of the lipid mixture in the aqueous medium was obtained by homogenization operating at temperatures above the mixture melting point (70–80 °C). This was subsequently subjected to high-frequency sonication treatment to reduce the particle size, thus obtaining a hot nano-emulsion which was then cooled at 4 °C during a second ultrasonic treatment to solidify the lipid phase thus obtaining the NLCs dispersion. All the obtained QRC-loaded dispersions appeared yellow, slightly cloudy, homogeneous, and easy to re-disperse. Neither microparticles nor crystals were detected by optical microscope in each preparation. The fresh dispersion of NLCs obtained from MIX 4 and MIX 5 were indicated as QRC-NLC-A and QRC-NLC-B, respectively, while the QRC-free nanoparticles (which turned out blank-opalescent) prepared from Blank 1 and Blank 2 mixtures were named NLC-A and NLC-B, respectively. The QRC-loaded NLCs were analyzed to determine the drug loading (DL%) and loading efficiency (LE%), being 4.98 ± 0.11% (DL) and 97.7 ± 0.08% (LE) for QRC-NLC-A, and 3.89 ± 0.12% (DL) and 88 ± 0.56% (EE) for QRC-NLC-B, respectively. Furthermore, both the QRC-loaded and the QRC-free NLCs, as well as the complete medium used for NLCs preparation (comprising the surfactants), were subjected to DLS and Z-potential measurements (Table 6). The particle size was not reported as Z-Average because the presence in all samples of surfactants in amount above of their CMC value allows micelles formation which leads to observing two populations, as confirmed by the analysis of the aqueous NLC-free medium (data not shown). This also results in misleading PDI values, although the experimental values are within the acceptable range for monodisperse systems (within 0.7) [63]. Finally, the Z-potential values indicate electrostatic repulsion that, alongside the steric stabilization derived from surfactants, both contributions make the dispersion thermodynamically stable, therefore avoiding nanoparticle aggregation phenomena [64].

According to the obtained data, the proposed QRC-NLC-A showed the best results in terms of mixture appearance, melting point, DL%, and LE%, as well as particle size, PDI and Z-potential. Moreover, as NLC-A showed higher particle size values, it is likely to notice that the presence of QRC allows to ameliorate nanoparticles creation. This is probably attributable to the very low melting point of the Blank 1 mixture that might promote the aggregation of particles through ripening processes, which can occur during the cooling and sonication steps. However, the introduction of QRC increased the mixture melting point, thus promoting the rapid solidification of the nano-drops already during the first moments of cooling, avoiding their aggregation. This hypothesis could also explain the particle size of NLC-B as well as of QRC-NLC-B, which have a melting point at least 10 degrees higher than Blank 1, thus reaching the solidification temperature at the beginning of the cooling process and avoiding any delay in the solidification step due to the energy of the sonication process.

**Figure 2:**
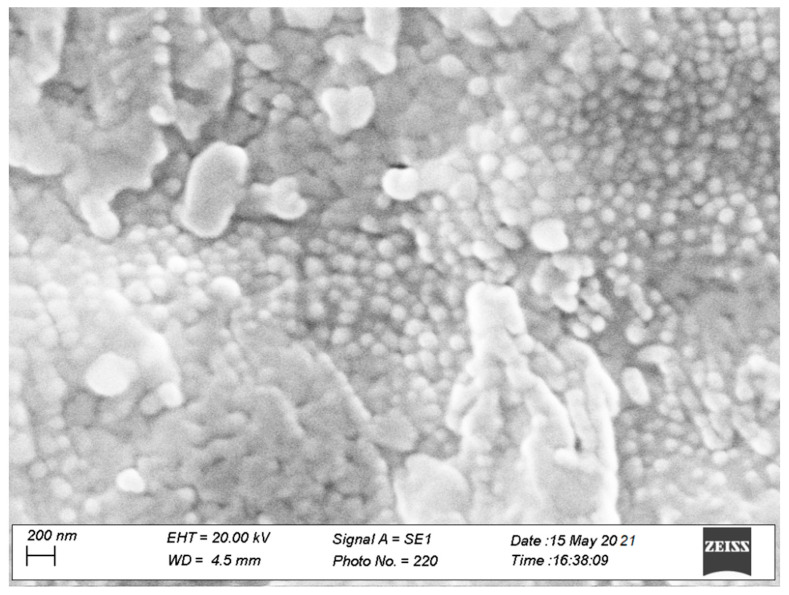
QRC-NLC-A surface morphology evaluated by SEM analysis; magnitude bar: 200 nm.

To confirm the DLS data, SEM images of the QRC-NLC-A sample were recorded, as reported in Figure 2.

The reported SEM image highlights the obtainment of spherical nanoparticles having a particle size consistent with the data obtained above, thus confirming the DLS analysis, as well as homogeneously distributed in size. Moreover, no crystalline structure of QRC was observed, suggesting that the drug was effectively encapsulated within the nanoparticles and surely in an amorphous state. Furthermore, nanoparticle agglomeration is observable. Likely, this should be due to the focused electron beam over the surface of the sample which can cause NLCs melting during sample analysis. According to these results, QRC-NLC-A was chosen as the best NLC formulation and they were similarly produced again by using a ciprofloxacin (CPX)-containing aqueous medium as dispersant phase. For the subsequent antimicrobial studies purpose, the corresponding QRC-free sample was also prepared. These nanodispersions were indicated as QRC-NLC-CPX and NLC-CPX (Table 7) and properly characterized.

The obtained results are satisfactory and confirm the possibility of employing the prepared QRC-loaded NLCs to obtain a multifunctional nanocomposite.

It was observed that the presence of the CPX in the aqueous phase affected the particle size, the PDI, and the Z-potential values by reducing all of them. Surprisingly, the greatest effect was observed when evaluating NLC-CPX particle size, likely due to CPX adsorption on the surface of nanoparticles resulting in preventing agglomeration, while the Z-Potential variation is probably related to the interaction of the CPX-salt with the charged NLCs surface [65].

### 3.3. Preparation and Characterization of the BioQ-CPX Nanocomposite

The development of a suitable polymeric network able to entrap the previously prepared nanoparticles between its chains through chemical or physical interactions is a key step to prepare a nanocomposite [66]. In this regard, the freeze-drying of a chitosan-based gel allows obtaining a polymeric matrix in which QRC-NLCs can be entrapped. Particularly, the final aim is to achieve a nanocomposite applicable in the post-extraction socket before the implant placement, able to promote the implant osteointegration by releasing active substances to enhance tissue healing and preventing microbial infections. Furthermore, it is necessary that the nanocomposite does not swell too much once applied in the target site, in order to avoid patient’s discomfort and any interference with the dental implant features.

Chitosan Liposan Ultra (CTS), as main hydrophilic network to embed the nanoparticles, was chosen for this aim. CTS is a physical mixture of partially deacetylated chitosan (about 90% w/w) and succinic acid, known in literature to have a low swelling degree in a weakly acidic environment [67]. Indeed, once the CTS-based nanocomposite is in vivo applied, it could interact with the physiological fluids and produce limited swelling due to the presence of the citrate buffer components from NLCs dispersion remained into the final formulation after lyophilization, being also able to reduce the environmental pH.

Moreover, CTS possesses suitable properties such as biodegradability, antimicrobial activity, low toxicity, and low immunogenicity [68]. Indeed, CTS’s osteoconductive behavior, structural similarities with glycosaminoglycans, and its well-known capability of inducing cell migration, adhesion, proliferation, and differentiation make it a fascinating biomaterial for tissue healing and regeneration [69]. Moreover, PVP K90, as supporting matrixing polymer, was added for its biocompatibility, bioadhesiveness, and ability to form hydrogels holding tissue compatibility and tissue-like consistency [70]. Finally, trehalose (THR) was inserted during the hydrogel preparation, as cryoprotectant, as it has already been reported to be particularly useful in preserving lipid-based composites subjected to freeze-drying process [62], contributing also to the long-term storage of the latter [71].

**Figure 3:**
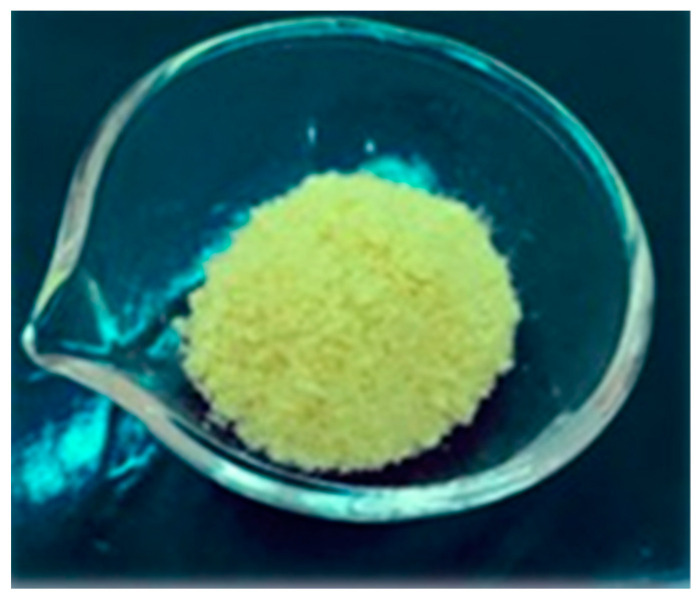
Appearance of the freeze-dried BioQ-CPX nanocomposite powder.

All the chosen components were suitable for freeze-drying and allowed to obtain a BioQ-CPX sample with proper morphological appearances. In particular, the resulting nanocomposite consisted of a soft and friable solid yellow powder, uniform in color and featuring a notable porous structure, created as a consequence of solvent removal during the freeze-drying process (Figure 3).

The suitability of such a nanocomposite material to be placed into the post-extraction socket is related to a wide variety of characteristics which should be accurately and adequately evaluated. The proposed material should by highly porous and low swellable in order to promote nutrients and oxygen passage while minimizing patient discomfort, respectively. In addition, for manufacturing purposes, it should be easy to handle and thus should not vary once in contact with the environmental moisture.

**Figure 4:**
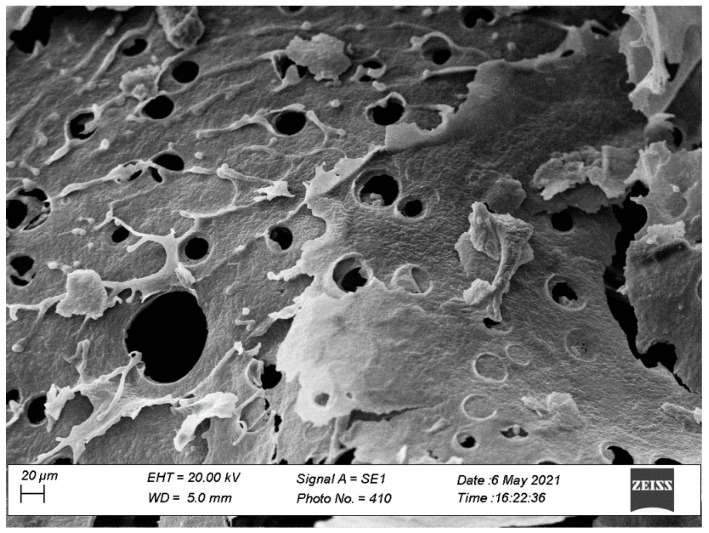
BioQ-CPX surface morphology evaluated by SEM analysis; magnitude bar: 20 µm.

The assessed BioQ-CPX porosity value was 94.1 ± 0.50%, which proves to be of great importance since materials having a porosity greater than 90% are ideal for tissue engineering applications, the osteoconductive properties of biomaterials being associated with over 75% porosity and pore sizes above 150 μm [44,63]. Indeed, a high porosity, an adequate pore size, and interconnections allow oxygen and nutrients to flow towards cells and exudate removal, influencing cell migration, infiltration, and angiogenesis phenomena [64,65]. The obtained results were suddenly confirmed by SEM analysis (Figure 4).

The drugs quantification into the nanocomposite was carried out on three randomly selected aliquots of each batch of BioQ-CPX, featuring DL% of 2.10 ± 0.03% and 3.50 ± 0.05% for QRC and CPX, respectively. These data demonstrated a homogeneous drug distribution as well as the reproducibility of the preparation method, as confirmed by the very low SE values.

**Figure 5:**
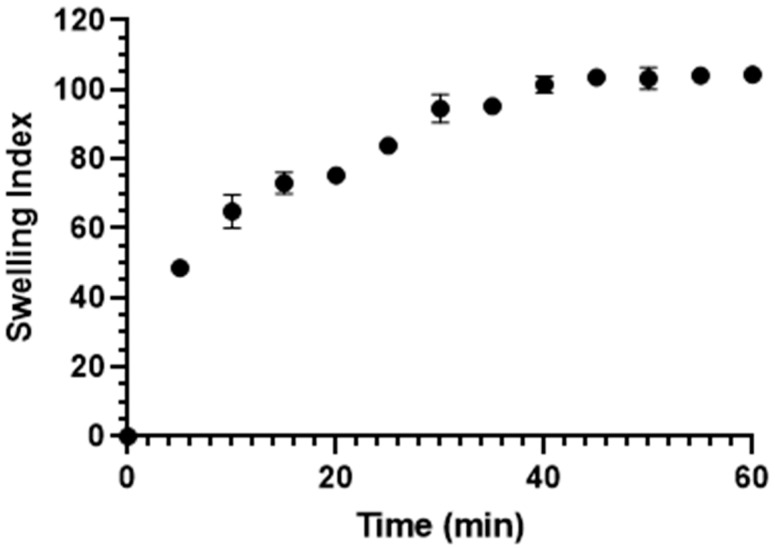
Swelling index as a function of time (min); mean ± SE (*n* = 3).

The ability of BioQ-CPX, to uptake physiological fluids was evaluated by swelling test, monitoring the weight increase over time. To overcome the difficulty of evaluating the sample as a powder, the latter was weakly compressed to obtain mini tablets having a diameter and thickness of 4.50 ± 0.02 mm and 2.50 ± 0.08 mm, respectively. Results showed a fast increase in weight within the first 5 min followed by a plateau which begins after 40 min when the maximum swelling index (100% of dry weight) is already observable (Figure 5).

It is known that the swelling behavior of chitosan is strongly dependent on the pH due to its chemical structure. The protonation of the amino groups occurring at low pH causes polymer chains repulsion and migration of counter ions resulting in a change in terms of osmotic pressure and increase in terms of water uptake and thus swelling. However, upon pH increase, these factors are reduced and lead to shrinking of the whole structure [72]. In this case, as the pH was set at 5.5 by using citrate buffer, the amino groups of chitosan and the carboxylic groups of the succinic acid were both in the ionized and not-ionized form. Thus, the interaction between positively charged chitosan moieties and negatively charged succinic acid molecules led to decrease of the polymer swelling ability [67]. This statement was confirmed by the maximum swelling degree of BioQ-CPX (100%) resulting 8-fold less than the not-modified chitosan at the same pH value, as reported in the literature [72].

**Figure 6:**
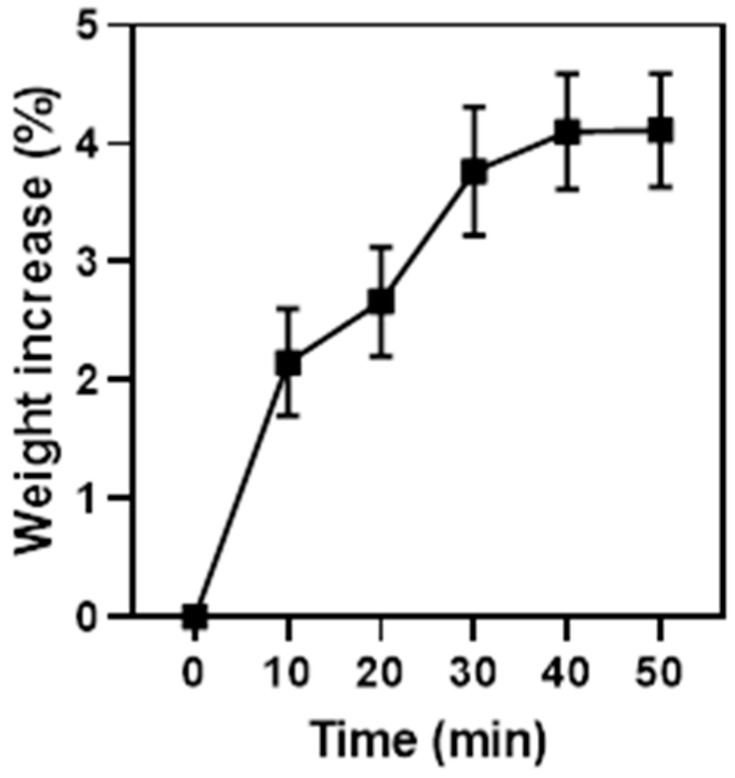
Hygroscopicity evaluation: BioQ-CPX weight increase percentage as a function of incubation time; mean ± SE (*n* = 6).

Another crucial parameter to be considered is powder hygroscopicity, which plays a key role in terms of handling as wet or high-moisture powders could be poorly flowing and difficult to store [73]. In particular, the ability of BioQ-CPX to absorb water from the surrounding environment was investigated and reported in terms of nanocomposite weight variation over time (Figure 6). As a result, the weight percentage increase at 60% RH reached a plateau after 40 min, therefore suggesting the establishment of an equilibrium with the environmental moisture. In any case, the maximum observed weight increase corresponded to 4.11 ± 0.48%, highlighting a slight hygroscopic behavior, which perfectly fits with the handling requirements.

As mentioned before, hygroscopicity strictly influences the powder storage. The production of pharmaceutical dosage forms as powder is a common way to protect drugs subjected to degradation as this process is generally enhanced by environmental moisture. QRC generally exhibits marked chemical instability, and its content in a dosage form could be dramatically reduced by both oxidation and degradation processes. The already observed poor hygroscopic aptitude of the system could thus play a key role in enhancing the stability of the nanocomposite itself.

### 3.4. Drugs Release Behaviors and Kinetic Evaluations

To achieve useful therapeutic effects, both the drugs embedded into the BioQ-CPX nanocomposite should be released. Since CPX and QRC do not share solubility properties, their release profiles and mechanism of drug discharge were investigated in vitro separately, using different approaches. This was due to their dissimilar chemical nature (logP_CPX_ = 0.28; logP_QRC_ = 1.50) leading to the need of different experimental set up in order to be able to observe the drug discharge behavior.

The mechanism governing the drug release process was studied by curve fitting the obtained experimental data to several empirical mathematic models, such as zero order, first order, Higuchi, and Korsmeyer–Peppas (Power Law), while other models that depend on a specific geometry (e.g., Hixson–Cromwell, Hopfenberg, and Peppas–Sahlin kinetics which respectively refer to tablets, thin films/cylinder/spheres, and DDS characterized by a defined aspect ratio) were considered not applicable in this case [74].

**Figure 7:**
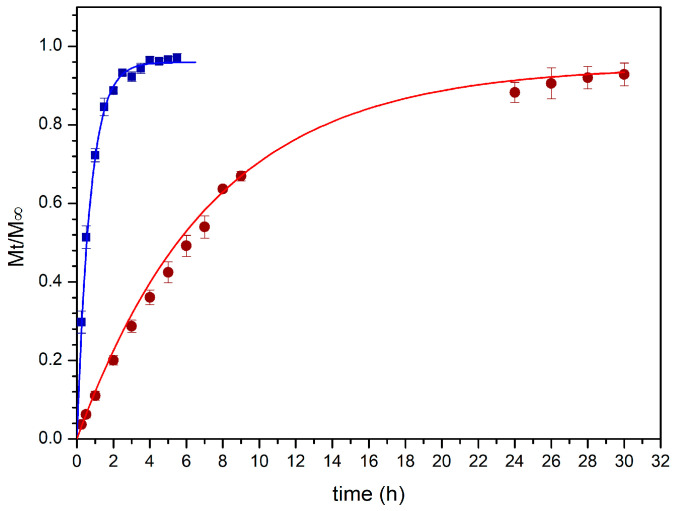
CPX dose fraction released from BioQ-CPX as a function of incubation time, by performing dialysis (■) and Franz cell (●) experiments. The First Order (solid lines) curve fitting is reported; means ± SE (*n* = 6).

Initially, dialysis technique was chosen to investigate CPX release by employing citrate buffer pH 5.5 amended with acetic acid as acceptor medium to assure sink conditions and a cellulose membrane (12–14 kDa) able to retain the nanocomposite powder, while avoiding any interference with CPX diffusion. However, due to the fast rate of CPX discharge in these experimental conditions (Figure 7), the information obtained left some doubts about the mechanism of the release process. Indeed, by curve-fitting the experimental data, a first order kinetic seems to occur. Particularly, 80% of CPX was released just after 1.5 h (Figure 7) indicating the prevailing of concentration as release driving force and not allowing to appreciate if other phenomena were involved.

Therefore, a new set of experiments was carried out using vertical Franz cells mounted with the same cellulose membrane (12–14 kDa) and employed as a bi-compartmental system. This experimental set up led to a reduction of the contact surface area between the formulation and the acceptor fluid as well as the amount of fluid which imbibed the nanocomposite, making it possible to observe a slowing down of the release trend from the first minutes of experiment (Figure 7) and correlate it with the swelling process, as observed in the specific experiments mentioned above. Once again, when the entire release behavior (30 h) was fitted to the mentioned mathematical models, the discharge process seemed to be governed by the CPX concentration gradient. This is likely due to CPX high water solubility which determines a high drug concentration into the meshes of the CTS-based matrix and thus in the donor compartment, according to the behavior of water-soluble drugs embedded in porous matrices (Figure 7).

**Figure 8:**
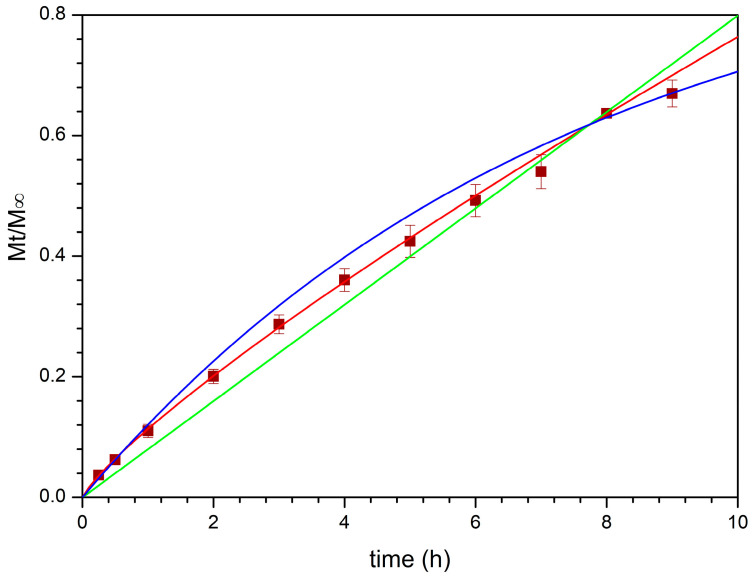
CPX dose fraction released from BioQ-CPX (■) as a function of incubation time, curve-fitted with the First Order (blue line), Zero Order (green line), and Korsmeyer–Peppas (red line) mathematical models; means ± SE (*n* = 6).

However, looking at the first part of the release profile (up to 9 h), the best fit having the regression coefficient value, R^2^, close to 1, was obtained with the Korsmeyer–Peppas model (Figure 8, Table 8), suggesting that more than one type of phenomenon of drug release was involved. Indeed, according to this model, the drug was transported via Fickian diffusion when the value of the parameter *n* was below or equal to 0.5, while the anomalous transport was supposed when *n* value was between 0.5 and 1.0 [75]. For CPX released from BioQ-CPX nanocomposite, the *n* value was 0.8280 ± 0.0125, suggesting a non-Fickian process due to a coupling of diffusion and swelling mechanisms in which the rate of the solvent diffusion and the polymeric relaxation possess similar magnitudes. Since the skeleton of the nanocomposite is mainly composed of CTS-based matrix wherein CPX is homogeneously distributed, drug release could be modulated by the swelling of the matrix, as well as the penetration of the medium in it. Indeed, CTS-based systems are usually characterized by drug sustained-release profiles in which the release rate depends on the pH of the dissolution media, being faster at an acidic pH than a neutral one [76].

To perform QRC release studies, a different experimental set up was tested. The rationale of the study was based on the hypothesis that, after the in vivo application into the post extraction socket, the nanocomposite could simultaneously release CPX as well as QRC-loaded NLCs as a whole. Indeed, QRC is a highly lipophilic molecule and consequently, it is unlikely to suppose its release from lipophilic nanoparticles to an aqueous, hydrophilic environment. It is plausible that the CTS matrix will release the NLCs, allowing their contact with the lipophilic domains of the buccal tissue and, subsequently, the NLCs will act as penetration enhancer for QRC, in order to promote and maximize its accumulation into the post extraction socket tissue.

As a consequence, the QRC release studies were reported as related to QRC amount as it is the only easily detectable component of the drug-loaded NLCs, but the proposed experiments were intended to evaluate both free QRC (if present) and QRC-loaded NLCs release from the surrounding CTS matrix. To this aim, transwell insert was selected as donor compartment by choosing an adequate pore size (0.45 µm) to enable NLC (particle size ≈300 nm) passage. To promote BioQ-CPX swelling and QRC release, the nanocomposite powder loaded inside the transwell insert was soaked with citrate buffer solution. A crucial point was related to the choice of the acceptor medium due to QRC poor solubility in many solvents, including lipophilic ones. While isopropyl myristate gave unsuccessful results, 1-octanol was a suitable acceptor fluid as it was able to efficiently solubilize QRC. During the experiments, it was observed that the total volume of donor medium did not vary as long as the experiment lasted, as well as no miscibility of fluids being visible thus confirming that the two compartmental fluids still remained separated and in close contact for the whole duration of the experiments.

**Figure 9:**
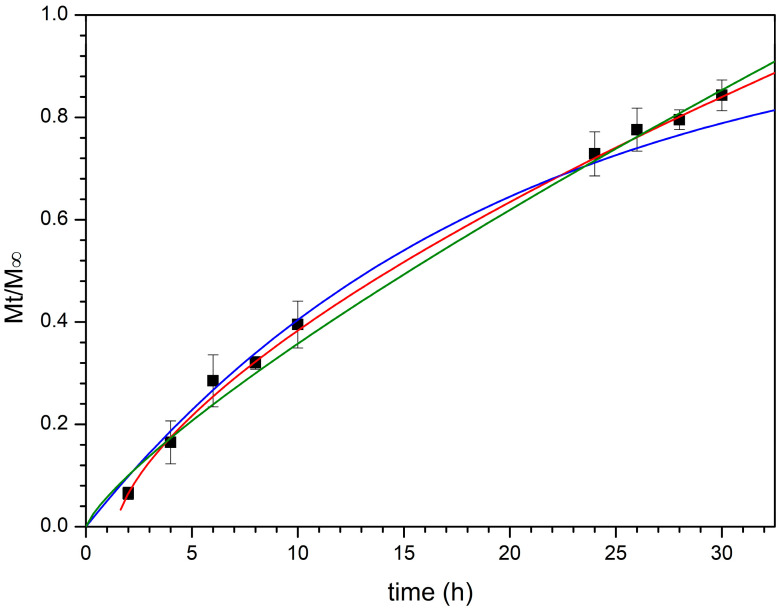
QRC dose fraction released from BioQ-CPX (■) as a function of incubation time, curve-fitted with the First Order (blue line), Korsmeyer–Peppas (green line), and Korsmeyer–Peppas with T_lag_ (red line) mathematical models; means ± SE (*n* = 6).

The QRC release profile is shown in Figure 9 and the previously mentioned mathematical models were all curve-fitted to the experimental data collected up to 30 h (Table 8) as in this case it is not possible to observe an evident plateau. At first glance, it can be noted that a good fit with the first order equation was not obtained, indicating how drug solubility significantly affects the release behavior.

As expected, the best curve-fitting was obtained by considering the Korsmeyer–Peppas model as the releasing matrix is always the CTS-based one, which controls the discharge of free QRC (if present) and QRC-loaded NLCs as a whole. In particular, the Korsmeyer–Peppas model opportunely modified to consider the T_lag_ is the most appropriate to describe the behavior of drug release. The obtained *n* value (0.6509 ± 0.0127) suggests, as already observed for CPX, a non-Fickian anomalous transport governed by both swelling and diffusion. In this case, the lag time extrapolated had a positive value indicating that a latency time occurs. This is likely due to nanoparticles diffusion process through the hydrophilic surroundings generated from the swollen CTS matrix to reach the acceptor medium and release QRC.

All the fitting parameters obtained by evaluating CPX and QRC release behaviors with the chosen mathematical models are summarized in Table 8. These results totally agree with the previously reported swelling studies in which emerged the ability of the nanocomposite to absorb water and swell over time until reaches a maximum swelling degree.

**$F=k_{KP}\times t^{n}$**

### 3.5. Ex Vivo Evaluation of Drugs Permeation/Penetration through/into Porcine Buccal Tissue

**Figure 10:**
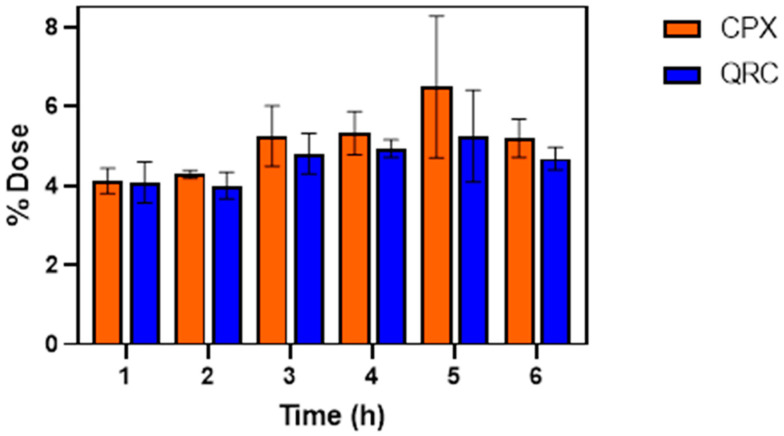
Percentage of CPX (orange) and QRC (blue) doses recovered into the porcine membrane at different time points (hours); means ± SE (*n* = 3). Significant differences between CPX and QRC groups (*p* < 0.05).

The ability of QRC and CPX to reach the bloodstream or accumulate into the tissue of the port-extraction socket was evaluated by performing ex vivo permeation/penetration studies, using the porcine buccal mucosa as a model membrane. Since the latter lacks keratinization and presents morphology, thickness, and lipid content similar to the human tissue, it represents the golden standard as animal model to test formulations to be applied in the oral cavity [77]. Moreover, due to its composition, the porcine buccal mucosa could also be used to mime the alveolus socket tissue. Thus, the donor chamber of a Franz cell was filled with citrate buffer (pH 5.5), to maintain stable QRC for the duration of the experiment. Instead, the acceptor chamber contained the same medium supplemented with acetic acid (0.1% v/v), Acys (0.002% w/v), and β-CD (3% w/v) to create sink condition for both drugs and inhibit their potential degradation as elucidated above. Indeed, the lack of solubility and/or stability of drugs could compromise the permeation process, thus producing misleading results. Since the nanocomposite aims to maintain an aseptic environment together with antioxidant effects in the tissues surrounding the post-extraction socket thanks to the sustained release of both CPX and QRC, the drugs’ ability to penetrate and accumulate into the porcine buccal mucosa was evaluated at different time intervals (from 1 to 6 h; Figure 10). As a result, the nanocomposite formulation was able to promote the drugs partitioning into the mucosa reaching in it an amount of about 4% of dose of both QRC and CPX already from the first hour from application. Then, they gradually continued to accumulate, reaching their maximum (about 5% of drugs dose) after the 5 h, therefore suggesting that membrane saturation occurs within this timeframe in the selected experimental conditions (e.g., contact surface area, membrane thickness).

Moreover, no permeation phenomena occurred as no detectable drug amount was observed in the acceptor compartment. These results perfectly fit with the desired characteristics of the prepared nanocomposite which could thus be efficiently placed into the post extraction socket so as to determine high drugs penetration and accumulation into the lipid domain of the alveolar tissue exerting locoregional effects while avoiding drug permeation and consequently drug loss into the bloodstream or systemic side effects.

### 3.6. Antioxidant Properties of BioQ-CPX Nanocomposite

Due to the well-known QRC susceptibility to degradation, it must be considered that several conditions involved in BioQ-CPX preparation such as pH, temperature, and sonication could cause QRC degradation, leading to a decrease in antioxidant properties of the resulting nanocomposite. For this reason, the antioxidant activity of BioQ-CPX, using BioCN-CPX (QRC-free) as a negative control and QRC (pure drug) as a positive one, was evaluated performing the DPPH assay.

**Figure 11:**
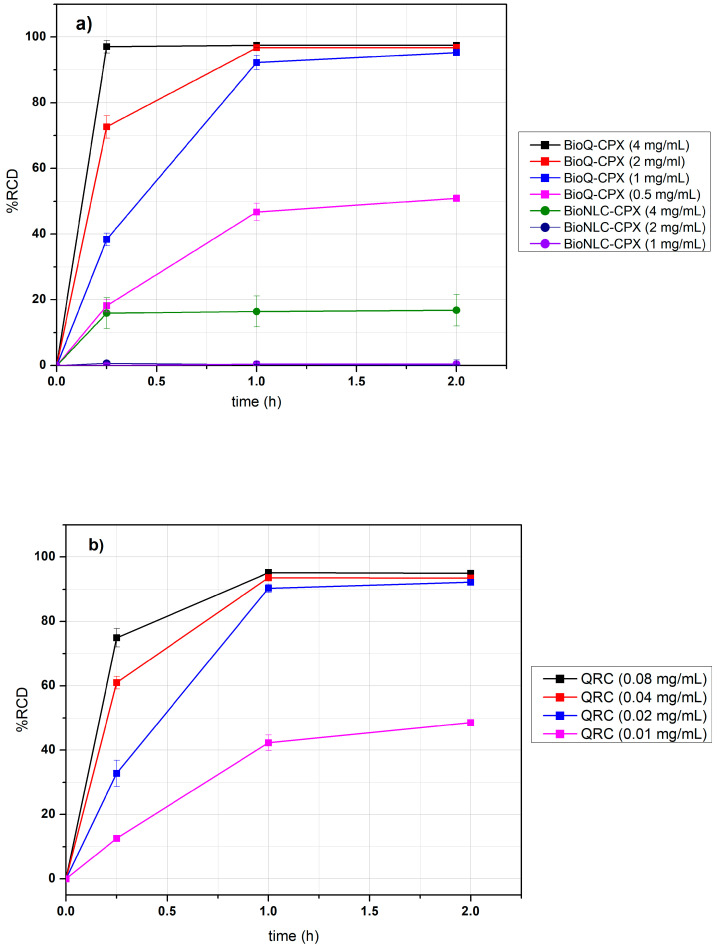
Percentage of residual concentration of DPPH (%RCD) radical form as a function of incubation time, analyzing: (**a**) BioQ-CPX at 4, 2, 1 and 0.5 mg/mL (squares) and BioNLC-CPX at 4, 2 and 1 mg/mL (circles) samples; (**b**) pure QRC at 0.08, 0.04, 0.02, and 0.01 mg/mL; means ± SE (*n* = 3).

Surprisingly, the results (Figure 11a) highlighted that the BioNLC-CPX possesses a weak antioxidant activity (%RCD = 16%) at the highest concentration analyzed, probably related to the presence of ACys and LIM into the formulation, while no evident antioxidant activity was observed at lower concentrations. As expected, the presence of QRC within the NLCs confers to BioQ-CPX a strong antioxidant power as a function of nanocomposite concentration. Indeed, 4 mg/mL of nanocomposite was able to reduce the whole DPPH radical form available within 15 min, while 2 mg/mL and 1 mg/mL, at the same time, reached 73 and 38 of %RCD, respectively. Furthermore, all the tested concentrations were able to entirely consume the DPPH radical form within 2 h. For the lowest BioQ-CPX concentration (0.5 mg/mL), the %RCD observed was 18% at 15 min, reaching the maximum of %RCD (50%) within 1 h, followed by a plateau (Figure 11a).

The QRC (pure drug) antioxidant activity (Figure 11b), evaluated at concentrations corresponding to QRC content in the nanocomposite samples, showed the same values of %RCD and trends after 1 and 2 h, while the scavenging activity was lower within 15 min incubation as compared to BioQ-CPX samples. These results suggest that no QRC degradation phenomena occurred during nanocomposite preparation and that synergistic scavenging effects among the nanocomposite components took place.

### 3.7. Antimicrobial and Anti-Biofilm Properties of BioQ-CPX Nanocomposite

**Figure 12:**
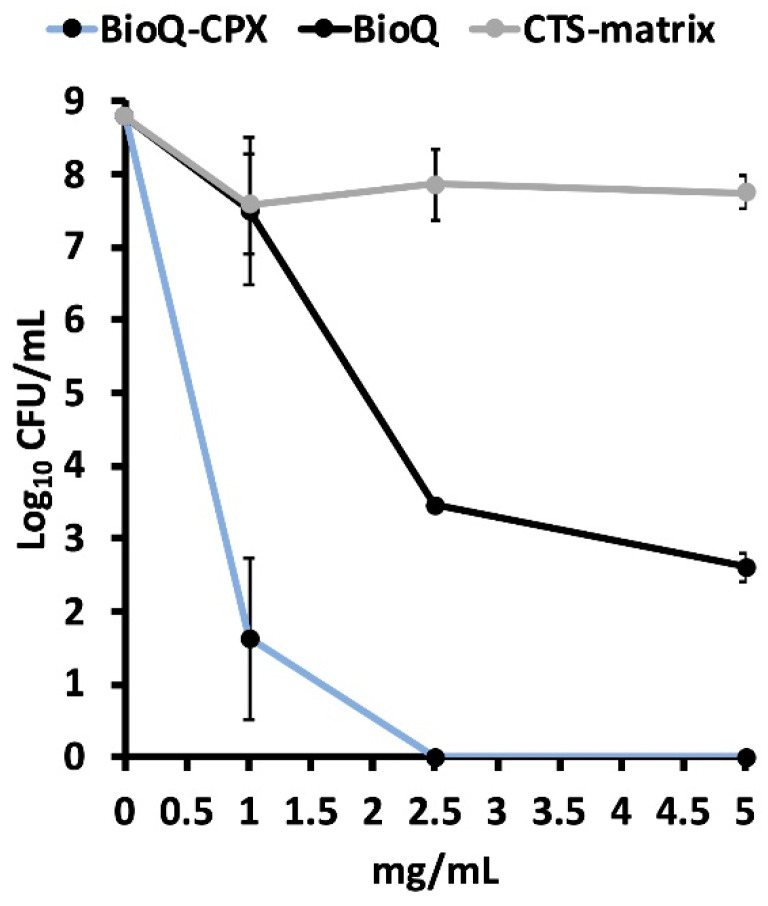
Antimicrobial activity of BioQ-CPX, BioQ, and CTS-matrix against *S. aureus* ATCC 25923 grown as planktonic cells as a function of applied concentrations (*n* = 3 ± SD).

In order to demonstrate the antimicrobial efficacy of CPX together with QRC when conveyed as nanocomposite, microbiological assays were performed. Obviously, only the amount of drug released from the dosage form will be able to exert antimicrobial activity, and CPX is known to be a fluoroquinolone active against Gram-positive bacteria such as *Staphylococcus aureus*. *S. aureus* species are common commensals of the oral microflora and transient members of the oral cavity [78]. Nevertheless, these microorganisms prevail over other microbes in older people and those affected by periodontal disease [79]. Moreover, it has been reported how the occurrence of staphylococci correlates with deeper peri-implant pockets featuring bleeding and may play a role in failing osseointegrated dental implants [14]. The overall picture is worsened by the high proficiency of staphylococci to form biofilms on permanent medical devices (i.e., dental implants; [80]). This aspect links to the detrimental role of these microbes in dental implant failure, and the spreading of nosocomial oral infections associated with it [81,82]. McCormack and colleagues [83] reported on how *S. aureus* species are frequently isolated from the oral cavity, 90% of clinical isolates being methicillin-sensitive (MSSA) *S. aureus* strains vs. 10% represented by methicillin-resistant (MRSA) ones. Thus, the oral cavity represents a reservoir of staphylococci capable of determining cross-infections and subsequent dissemination to other anatomic sites, especially if affected by surgery. Given these premises, the indicator pathogen MSSA *S. aureus* ATCC 25923 strain was chosen as Gram-positive bacterium model to be challenged—under planktonic growth mode—against BioQ-CPX, BioQ, and the blank CTS-matrix (Figure 12) to attribute the antimicrobial efficacy to the complete nanocomposite rather than to the same without CPX or without CPX and NLC-QRC. As a result, the kill curve highlights how 1 mg/mL of the complete formulation (BioQ-CPX) was sufficient to achieve a drastic logarithmic (log) reduction (ca. seven units) of the *S. aureus* CFU/mL. An increased dose of BioQ-CPX up to 2.5 mg/mL determined a 100% killing effect. In contrast, the highest concentration (5 mg/mL) of BioQ, although resulting in 6 log-units reduction of the CFU/mL evaluated, failed in completely suppressing biomass resistance to the challenge (Figure 12).

**Figure 13:**
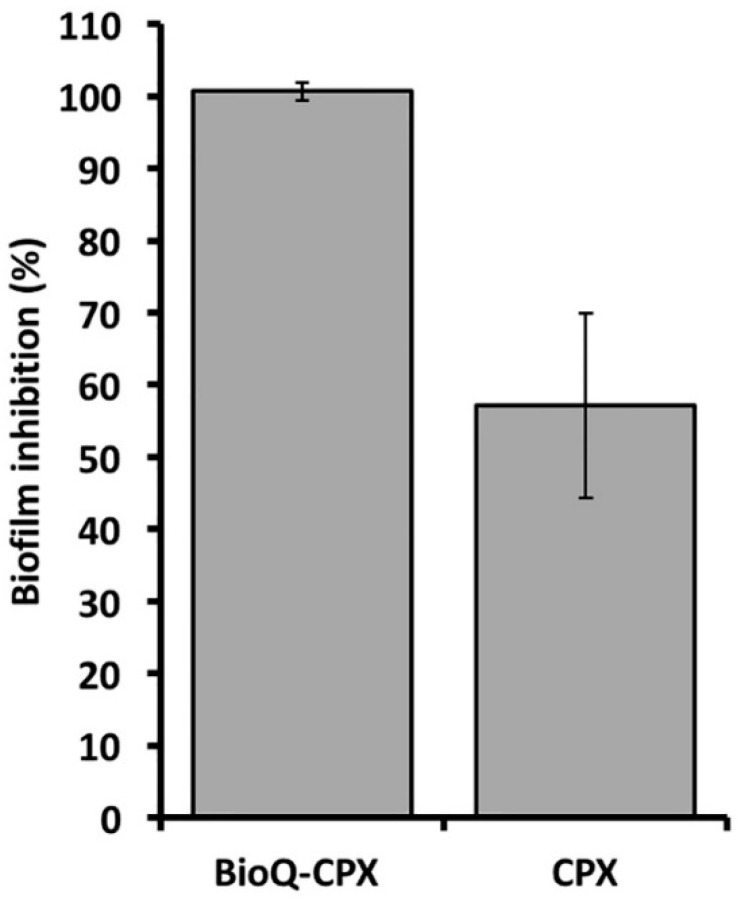
Antimicrobial activity of BioQ-CPX and CPX (as a reference) supplied as disks (5 mg) against *S. aureus* cells growing as a biofilm (*n* = 3 ± SD). Significant differences between BioQ-CPX and CPX groups (*p* < 0.05).

A reasonable explanation can rely on the high minimal inhibitory concentration (MIC) of phenolic compounds (i.e., the flavonoid quercetin) against both Gram-positive and -negative bacteria when these bioactive molecules are administered alone. For instance, the inhibitory effect of QRC against both MSSA and MRSA strains occurs in the range comprises between 0.1 and 0.6 mg/mL [84], being in line with the amount of QRC—ca. 0.1 mg/mL for the highest concentration tested—present in both BioQ-CPX and BioQ nanocomposite formulations. Yet, the killing effect of BioQ might rely on the successful drug delivery and slow release of QRC elicited by lipid nanoparticles in which QRC is loaded, therefore enhancing its targeting at the cell level. Thus, the strength of the BioQ-CPX formulation could depend on a synergistic effect between QRC and CPX. On the one hand, the former can act (i) breaking down the structure of the cytoplasmic membrane, leading to the loss of its integrity, increasing its permeability, and dissipating the membrane potential [85,86], (ii) interfering with the synthesis of the peptidoglycan layer, (iii) inhibiting the nucleic acid synthesis, and (iv) impairing the energy transport systems [87]. On the other hand, the entrance of CPX within the bacterial cytosol would enhance, facilitating the fluoroquinolone action at this level (i.e., impairment of DNA replication), potentially allowing for the decrease of the antibiotic dosage using such a formulation, thanks to the combined effect of natural and synthetic compounds. It is worth mentioning that, although the highest amount (ca. 0.1 mg/mL in the BioQ-CPX) of CPX did prevent bacterial growth (data not shown), the sole administration of this antibiotic would not serve the purpose of this study, which relies on the slow local delivery and residency of bioactive molecules in the post-extraction socket prior dental implant placement. Finally, the CTS matrix did only exert a slight decrease of the CFU/mL at its lowest concentration, afterward acting as a bacteriostatic agent against *S. aureus* cells (Figure 12, an aspect that is likely due to CTS concentration. Indeed, the main mechanism for CTS antimicrobial activity relies on its polycationic nature that determines CTS binding to negatively charged moieties present in the bacterial cell surface, compromising cell wall and membrane integrity. This allows for CTS attachment to DNA upon its entry at the cytosol level, impairing the replication of the latter, which ultimately causes cell death [88]. Conversely, high CTS concentrations may coat the bacterial cell surface through CTS protonated moieties preventing the leakage of cell components, repelling bacterial cells from each other, and avoiding agglutination phenomena [89], thus determining bacterial adaptation and survival upon increasing the CTS concentration (Figure 12). To further sustain the applicability of the BioQ-CPX formulation for maintaining aseptic conditions in peri implant-surrounding tissues, the capability of this nanocomposite of preventing infections was tested against *S. aureus* cells growing as a biofilm (Figure 13), as the latter represents the real threat for patients undergoing dental surgical procedures. In this regard, most bacteria feature the innate ability to colonize a vast array of surfaces as a biofilm, which is an aspect of collective concern for human health, particularly when biofilms contaminate tools that must be sterile, such as dental hygiene equipment and medical devices [90]. Moreover, biofilm cells are surrounded and protected by an extracellular polymeric substance (EPS) that overall provides stability to the microbial community and acts as a shield against most biocides [91], as also highlighted by CPX failure in totally inhibiting (ca. 55% inhibition) *S. aureus* biofilm formation (Figure 13).

Nevertheless, BioQ-CPX could completely prevent the growth of the staphylococcal biofilm, likely due to the collateral action of QRC present in the formulation. In this regard, it was previously reported that QRC can act as anti-quorum sensing—the cell to cell communication system behind the regulation of important genes coding for virulence factors, biofilm formation, and antibiotic resistance—compound against the Gram-negative pathogen *Pseudomonas aeruginosa*, interfering with coordination events occurring within the microbial community and regulating its density of population [92,93]. Furthermore, Mu and colleagues [94] reported on the QRC capability of decreasing (i) the amount of EPS production by *S. epidermidis* and its composition, (ii) the cell surface hydrophobicity (i.e., an important factor on which relies the cell attachment), and (iii) the upregulation of the *icaR* gene encoding for the transcriptional repressor of the *ica* locus (responsible for cell-to-cell adhesion [95], determining the downregulation of the latter and causing the reduction of biofilm formation. Thus, although the limitation of these antibacterial studies is that they were only conducted against *S. aureus*, the promising obtained results allow hypothesizing that the presence into the nanocomposite of QRC-NLCs might support a multitarget activity of BioQ-CPX against other bacteria strains, even though this aspect needs to be further demonstrated.

Regardless, the technological approach combining QRC, encapsulated in lipid nanocarriers, and CPX, entrapped in CTS-matrix, generated a nanocomposite that successfully releases bioactive molecules and prevents the staphylococcal biofilm formation in the surrounding of implant placing. This result is further corroborated by the low amount of QRC and CPX (ca. 100 µg/mL each) present within the BioQ-CPX as compared to the anti-biofilm effect that can be achieved by solely using the antibiotic agent.

### 3.8. Stability of BioQ-CPX Nanocomposite

To evaluate the BioQ-CPX capability in preserving QRC from light exposure, electron paramagnetic resonance (EPR) spectroscopy was used. EPR spectroscopy is a technique employed to detect and study paramagnetic molecules or materials that contain unpaired electrons such as antioxidant compounds. By this technique, information can be obtained about the interactions with other nuclei around unpaired electron, providing evidence on the structure, by parameters calculation from the EPR spectrum such as amplitude peak–peak (H_pp_), linewidths (ΔB_pp_), and g-factors. In particular, amplitude H_pp_ proportionally increase by increasing of paramagnetic center concentration of the sample, ΔB_pp_ and g-factors depend on the molecular structure of the sample and on the magnetic interactions in the chemical units.

Preliminarily, the paramagnetic center of QRC was characterized before and after UV radiations exposure. The EPR spectrum of not irradiated QRC (Figure S1, continuous line) showed a single peak; the amplitude H_pp_ of singlet was calculated by summing in absolute value the maximum and the minimum of the peak, normalized to mass and corresponding to 0.054 u.a. The ΔB_pp_ was width of single peak and corresponded to 19 G. The g-factor of the peak was calculated using the following equation:

$$g\;=\frac{\mathrm{hv}}{\mu_{B}B}$$

where h = Planck constant, ν = microware frequency, μ_B_ = Bohr Magneton, and B = magnetic field and it corresponds to 2.0080.

The EPR spectrum of 20 min-irradiated QRC (Figure S1, dashed line) using the 50 W lamp also showed a singlet. The ΔB_pp_ and g-factor were calculated and corresponded to same value of not irradiated QRC. The H_pp_ value normalized to mass corresponding to 1.018 u.a. These results indicated that the UV radiation caused a concentration increase of the same paramagnetic center resulting in H_pp_ value, but no creation of news paramagnetic centers on QRC was highlighted indicating that no new chemical species were formed.

Afterwards, EPR spectra of BioQ-CPX not irradiated and UV irradiated (50 W lamp) for 20 min were recorded (Figure S2). Both EPR spectra showed a singlet; the ΔB_pp_ and g-factor of both signals were calculated and corresponded to the same value of pure QRC; the paramagnetic center of QRC was not modified during the BioQ-CPX preparation, and the EPR signal of BioQ-CPX was not due to the excipients of the nanocomposite. The low value of H_pp_ of both peaks was probably related to QRC amount into the nanocomposite (≈ 2.1%) rather than to a reduction in concentration of paramagnetic centers.

**Figure 14:**
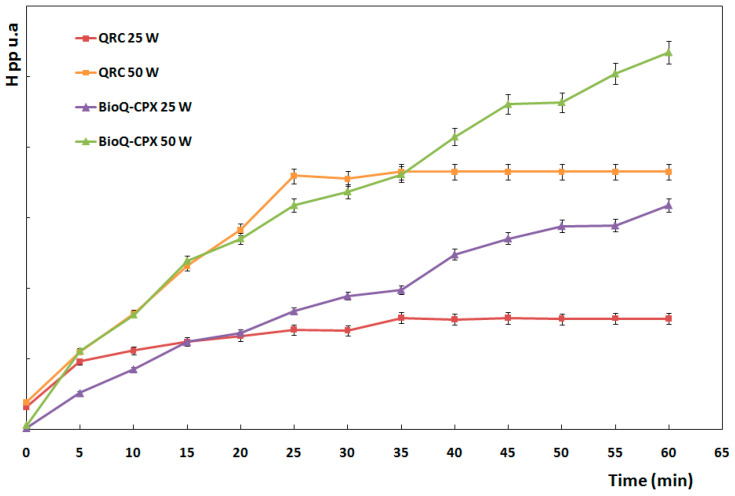
H_pp_ values as a function of UV irradiation time (25 W and 50 W lamps) for pure QRC (squares) and BioQ-CPX nanocomposite (triangles); means ± SE (*n* = 3).

To evaluate if QRC photosensitivity can be reduced by incorporating QRC in the nanocomposite, samples of QRC and BioQ-CPX were irradiated over time and EPR spectra were acquired every 5 min during UV irradiation. Figure 14 shows the relationship between H_pp_ normalized to QRC mass and time of UV exposure by 25 W and 50 W lamps, respectively.

After 5 min of QRC (pure drug) irradiation, the intensity values of H_pp_ obtained by using 25 W or 50 W lamp were not significantly different; after a linear trend increasing up to 25 min of exposure, showing a higher slope when the QRC was irradiated by 50 W lamp, a plateau was reached in both cases, indicating that the concentration of the available paramagnetic centers no longer increased, i.e., the amount of QRC available to oxidize (antioxidant power) was exhausted.

In the same conditions, the intensity of the EPR signal of BioQ-CPX vs. UV irradiation time showed an increasing monotonous trend, with a higher slope for sample irradiated by 50 W lamp, as seen for pure QRC. In this case, no plateau was observed until 60 min, highlighting the progressive increase in paramagnetic centers concentration, which means that the QRC loaded into the nanocomposite still possesses antioxidant properties. Consequently, it is likely to highlight the actual possibility of preserving QRC activity by embedding this molecule into the proposed nanocomposite.

**Figure 15:**
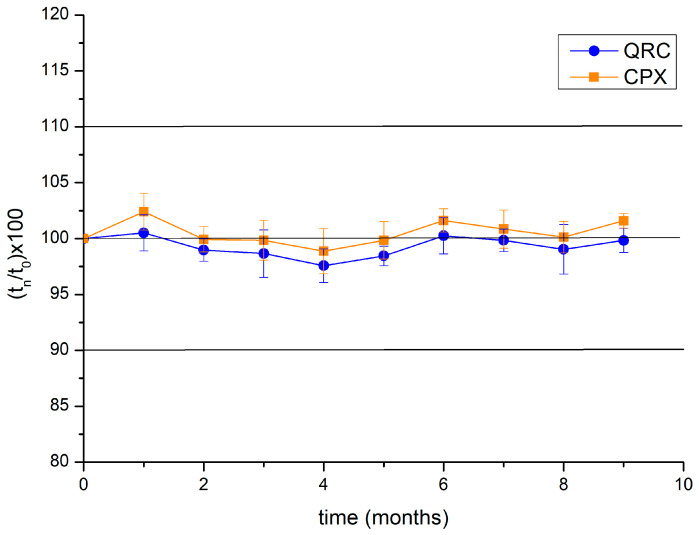
Percentage of CPX (●) and QRC (■) content variation into the BioQ-CPX as a function of time (months); means ± SE (*n* = 3).

The ability of the BioQ-CPX nanocomposite in preventing drugs degradation was studied at room temperature and under natural light exposure by monitoring QRC and CPX content up to nine months (Figure 15).

The results showed that the percentage of content variation for both drugs in the nanocomposite was between 95 and 105%, confirming the effectiveness of the formulation to preserve the chemical stability of QRC and CPX over time.

These stability data, together with those previously obtained (e.g., hygroscopicity studies), confirmed that the proposed material is highly handy as well as easy to store for prolonged period.

## 4. Conclusions

Immediate implant placement has lately been proposed as an innovative approach to restore missing teeth. In this work, a new drug delivery system in the form of a nanocomposite was accurately designed to be directly applied into the fresh post-extraction socket before the restorative treatment. The research work firstly led to developing QRC-loaded nanostructured lipid carriers by a coupled homogenization and high frequency ultrasound process. By choosing suitable solid and liquid lipids, such as Labrasol^®^ and GMS, combined in appropriate ratio to encapsulate 5% (w/w) of QRC, nanoparticles with suitable particle size, PDI, and Z-potential values were obtained. These were then successfully embedded by a freeze-drying process into a CTS-based CPX-loaded hydrophilic matrix to obtain the BioQ-CPX nanocomposite as a powder. The proposed formulation was useful for the starting purpose as it exhibited adequately porosity, slight hygroscopicity, and ability to stabilize and preserve the two encapsulated drugs for prolonged time. According to in vitro release and permeability studies, BioQ-CPX nanocomposite was able to control drugs discharge in a reproducible manner and promote drugs accumulation into mucosal tissue simulating the alveolar socket one. Moreover, the nanocomposite expressed its effectiveness in terms of antioxidant power more than QRC pure thanks to synergistic scavenging effects among all components. Furthermore, the observed antibacterial activity against *Staphylococcus aureus* proved that the new drug delivery system was able to make the drugs bioavailable in situ, even causing the reduction of biofilm formation respect to CPX alone. Although the microbiological evidence is limited against *S. aureus* as bacterium model, the obtained findings confirmed that an innovative delivery strategy focused on the combination of nanovehicles (NLCs for QRC administration) and polymeric matrix systems (CTS-based matrix for CPX administration) could be a winning approach to obtain the beneficial loco-regional effects of drugs in preventing bacterial infections, biofilm formation and oxidative stress damages, enhancing tissue healing.

In light of the obtained results, since the success of implant osseointegration mostly depends on the health of the surrounding bone, the designed system could surely provide a suitable environment for dental implant placement, supporting the single-stage dental restorative approach.

## Figures and Tables

**Table 1:** Percentage composition (w/w) of the lipid mixtures.

Sample	QRC (%)	Labrasol^®^ (%)	GMS (%)	GA (%)
MIX 1	5	46	46	3
MIX 2	5	42.5	42.5	10
MIX 3	5	45	45	5
MIX 4	5	24	68	3
MIX 5	5	24	66	5
Blank 1	0	25	72	3
Blank 2	0	25	70	5

**Table 2:** List of prepared nanocomposites.

NLC Samples	Nanocomposite Samples
QRC-NLC-A	BioQ
NLC-A	BioNLC
QRC-NLC-CPX	BioQ-CPX
NLC-CPX	BioNLC-CPX

**Table 3:** QRC solubility (mg/mL) ± SE in various aqueous media at 37 ± 0.5 °C.

Aqueous Media	Solubility (mg/mL)
Citrate buffer pH 5.5/ACys plus β-CD 2% w/v	0.083
Citrate buffer pH 5.5/ACys plus β-CD 3% w/v	0.1033
Citrate buffer pH 5.5/ACys plus DMSO 10% v/v	0.029
Water pH 7	0.002 [52]

**Table 4:** Visual evaluation of QRC dissolution in selected liquid lipids.

Liquid Lipid Excipient	Appareance
Labrasol^®^	Clear
Capryol PGMC^®^	Opalescent
Plurol^®^	Opalescent
Maisine^®^	Opalescent
Labrafil^®^ M 1944 CS	Opalescent
PEG-18 G/C	Slightly Opalescent

**Table 5:** Evaluation of the tested lipid mixtures in terms of melting temperature range and appearance of the melted mass.

Sample	Melting Temperature (°C)	Appearance at 120 °C	Components Ratio (a:b:c:d)
MIX 1	48–40	Slightly cloudy	5:46:46:3
MIX 2	55–60	Cloudy and brown	5:42.5:42.5:10
MIX 3	45–50	Slightly cloudy	5:45:45:5
MIX 4	50–55	Limpid	5:24:68:3
MIX 5	55–60	Limpid	5:24:66:5
Blank 1	30–35	Limpid	0:25:72:3
Blank 2	40–45	Limpid	0:25:70:5

a: QRC; b: Labrasol^®^; c: GMS; d: GA.

**Table 6:** Particle size (nm), PDI, and Z-potential (mV) ± SE of NLCs samples.

Sample	Particle Size (nm)	PDI	Z-Potential (mV)
QRC-NLC-A	337.8 ± 61.4	0.546	−24.8 ± 5.3
QRC-NLC-B	399.2 ± 138.1	0.608	−16.1 ± 4.5
NLC-A	878.3 ± 177.1	0.353	−26.4 ± 6.1
NLC-B	332.1 ± 96.8	0.350	−29.2 ± 5.3

**Table 7:** Particle size (nm), PDI, and Z-potential (mV) ± SE of QRC-NLC-CPX and NLC-CPX samples.

Sample	Particle Size (nm)	PDI	Z-Potential (mV)
QRC-NLC-CPX	292.5 ± 78.5	0.388	−7.23 ± 9.3 V
NLC-CPX	358.16 ± 113	0.301	−13.9 ± 4.9 V

**Table 8:** Mathematical models fitted to the experimental QRC and CPX release curves: calculated fitting parameters and square of correlation coefficient (R^2^).

Mathematical Models	CPX Fitting Parameters *			QRC Fitting Parameters **		
	*k*	*n*	*r* ^2^	*k*	*n*	*r* ^2^
Zero-Order **$F=k_{0}\times t$**	0.079		0.99464	0.02997		0.96113
First-Order ** $F=F_{max}\times \left(1-e^{-k_{1}\times t}\right)$	0.13587		0.99767	0.05179		0.98912
Higuchi $F=k_{H}\times t^{0.5}$	null		null	null		null
Korsmeyer-Peppas $F=k_{KP}\times t^{n}$	0.1134	0.8280	0.99964	0.0578	0.79146	0.99021
Korsmeyer-Peppas with T*_lag_* **$F=k_{KP}\times {\left(t-T_{lag}\right)}^{n}$**	0.11131 (T*_lag_* = −0.031)	0.8293	0.99959	0.0947 (T*_lag_* =1.440)	0.6509	0.99934

* Fitting with experimental data up to 9 h. ** Fitting with experimental data up to 30 h. F = fraction of released drug in time t; F_max_ = maximum fraction of the drug released at infinite time; k_1_ = first-order release constant, k_H_ = Higuchi release constant; k_KP_ = release constant based on structural characteristics of the drug-dosage form; *n* is the diffusional exponent indicating the drug-release mechanism plotting the fraction released (Mt/M∞) vs. time (hours).

## Data Availability

All data available are reported in the article.

## Supplementary Materials

The following are available online at https://www.mdpi.com/article/10.3390/pharmaceutics13122072/s1, Figure S1: EPR spectra of QRC not irradiated (continuous line) and UV irradiated (50 W) for 20 min (dashed line). The Hpp and ΔBpp parameters are shown, Figure S2: EPR spectra of BioQ-CPX not irradiated (continuous line) and UV irradiated (50 W) for 20 min (dashed line).
