# Supplementary material for: Lipid Nanocarriers-Loaded Nanocomposite as a Suitable Platform to Release Antibacterial and Antioxidant Agents for Immediate Dental Implant Placement Restorative Treatment
Source: Pharmaceutics. 2021 Dec 3;13(12):2072. doi: 10.3390/pharmaceutics13122072 (PMC8706998; doi:10.3390/pharmaceutics13122072)
Supplement: Supplementary file 1 [file pharmaceutics-13-02072-s001.zip › pharmaceutics-1472835-supplementary.pdf]

pharmaceutics

MDPI

Supplementary Materials: Lipid Nanocarriers-Loaded Nanocomposite as a Suitable Platform to Release Antibacterial and Antioxidant Agents for Immediate Dental Implant Placement Restorative Treatment

Giuseppe Angellotti, Alessandro Presentato, Denise Murgia, Giulia Di Prima, Fabio D'Agostino, Maria Cristina D'Oca, Amalia Giulia Scarpaci, Rosa Alduina, Giuseppina Campisi and Viviana De Caro

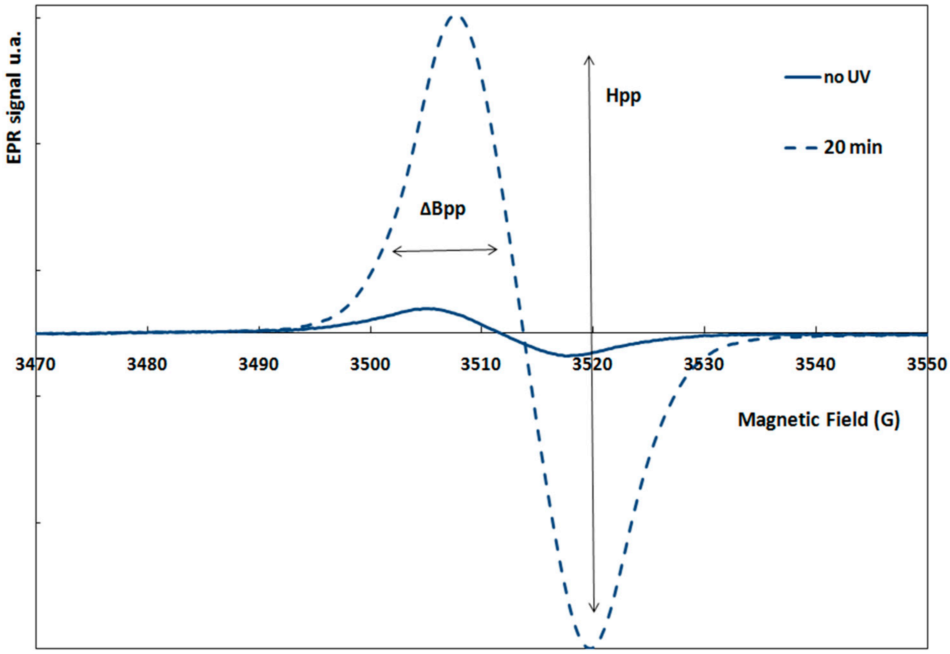

Figure S1. EPR spectra of QRC not irradiated (continuous line) and UV irradiated (50 W) for 20 min (dashed line). The H_{pp} and ΔB_{pp} parameters are shown.

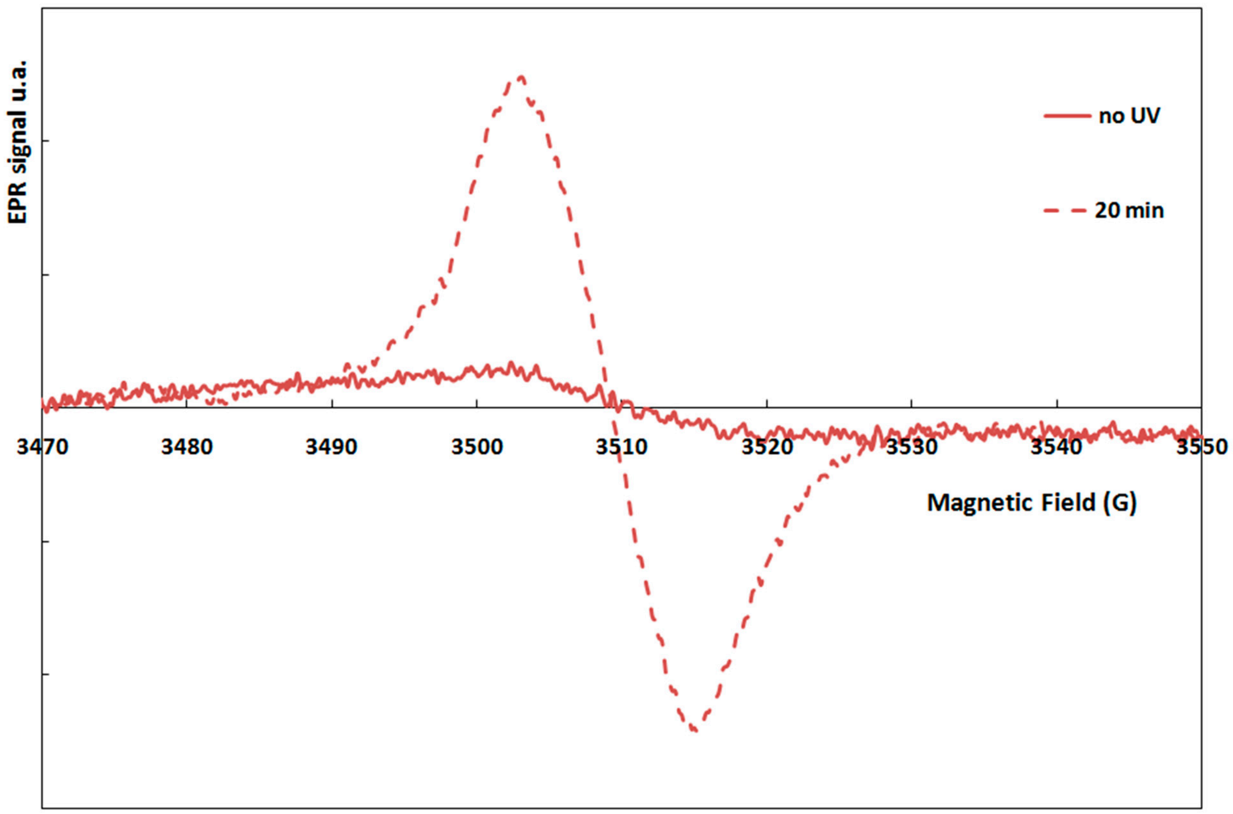

Figure S2. EPR spectra of BioQ-CPX not irradiated (continuous line) and UV irradiated (50 W) for 20 min (dashed line).
